# Effect of a Skin Self-monitoring Smartphone Application on Time to Physician Consultation Among Patients With Possible Melanoma: A Phase 2 Randomized Clinical Trial

**DOI:** 10.1001/jamanetworkopen.2020.0001

**Published:** 2020-02-26

**Authors:** Fiona M. Walter, Merel M. Pannebakker, Matthew E. Barclay, Katie Mills, Catherine L. Saunders, Peter Murchie, Pippa Corrie, Per Hall, Nigel Burrows, Jon D. Emery

**Affiliations:** 1 The Primary Care Unit, Department of Public Health and Primary Care, University of Cambridge, Cambridge, United Kingdom; 2 Department of General Practice and the Centre for Cancer Research, Faculty of Medicine, Dentistry, and Health Science, Victorian Comprehensive Cancer Centre, University of Melbourne, Australia; 3 Institute of Applied Health Science, Centre of Academic Primary Care, University of Aberdeen, Aberdeen, United Kingdom; 4 Cambridge University Hospitals NHS Foundation Trust, Cambridge, United Kingdom

## Abstract

**Importance:**

Melanoma is among the most lethal skin cancers; it has become the fifth most common cancer in the United Kingdom, and incidence rates are rising. Population approaches to reducing incidence have focused on mass media campaigns to promote earlier presentation and potentially improve melanoma outcomes; however, interventions using smartphone applications targeting those with the greatest risk could promote earlier presentation to health care professionals for individuals with new or changing skin lesions.

**Objective:**

To study the effect of a commercially available skin self-monitoring (SSM) smartphone application among individuals with increased risk of melanoma on their decision to seek help for changing skin lesions.

**Design, Setting, and Participants:**

This phase 2 randomized clinical trial was conducted in 12 family practices in Eastern England between 2016 and 2017. A total of 238 participants, aged 18 to 75 years and with an increased risk of melanoma, were identified using a real-time melanoma risk assessment tool in family practice waiting rooms. Analysis was intention to treat. Participants were observed for 12 months, and data analysis was conducted from January to August 2018.

**Intervention:**

The intervention and control groups received a consultation with standard written advice on sun protection and skin cancer detection. The intervention group had an SSM application loaded on their smartphone and received instructions for use and monthly self-monitoring reminders.

**Main Outcomes and Measures:**

The coprimary outcomes were skin consultation rates with family practice physicians and patient intervals, measured as the time between noticing a skin change and consulting with a family practice clinician. Follow-up questionnaires were sent at 6 and 12 months, and consultation rates were extracted from family practice records. Secondary outcomes included skin self-examination benefits and barriers, self-efficacy for consulting without delay, perceived melanoma risk, sun protection habits, and potential harms.

**Results:**

A total of 238 patients were randomized (median [interquartile range] age, 55 [43-65] years, 131 [55.0%] women, 227 [95.4%] white British; 119 [50.0%] randomized to the intervention group). Overall, 51 participants (21.4%) had consultations regarding skin changes during the 12 months of follow-up, and 157 participants (66.0%) responded to at least 1 follow-up questionnaire. There were no significant differences in skin consultation rates (adjusted risk ratio, 0.96; 95% CI, 0.56 to 1.66; *P* = .89), measures of SSM (adjusted mean difference, 0.08; 95% CI, −0.83 to 1.00; *P* = .86), or psychological harm (eg, Melanoma Worry Scale: adjusted mean difference, −0.12; 95% CI, −0.56 to 0.31; *P* = .58).

**Conclusions and Relevance:**

In this study, recruitment, retention, and initial delivery of the intervention were feasible, and this research provided no evidence of harm from the SSM smartphone application. However, no evidence of benefit on skin self-examination or health care consulting was found, and there is no reason at this stage to recommend its implementation in this population at increased risk of melanoma.

**Trial Registration:**

isrctn.org Identifier: ISRCTN16061621

## Key Points

**Question:**

Does a smartphone application that prompts monthly skin self-monitoring result in individuals with increased risk of melanoma having more timely or more frequent family practice consultations for skin changes?

**Findings:**

In this phase 2 randomized clinical trial of 238 participants, there were no significant differences in skin consultation rates, measures of skin self-monitoring, or psychological harm.

**Meaning:**

Because there was no evidence that a smartphone application increased skin self-examination or health care consulting rates among a family practice population at increased risk of melanoma, its implementation is not yet recommended.

## Teaser

This phase 2 randomized clinical trial studies the effect of a commercially available skin self-monitoring smartphone application among individuals with increased risk of melanoma on their decision to seek help for changing skin lesions.

## Introduction

Melanoma is among the most lethal forms of skin cancer.^1^ While it accounts for less than 5% of all cutaneous malignant neoplasms worldwide, melanoma is responsible for most skin cancer deaths.^2^ Internationally, melanoma has among the fastest rising incidence rates of any cancer.^3^ In the United States, during the 7 years from 2009 to 2016, raw incidence rates per 100 000 residents climbed from 22.2 to 23.6, with an estimated 76 380 new cases in 2016.^4^ In England, in the decade from 2007 to 2016, age-standardized incidence increased by 37%, with 13 748 new cases and 1937 deaths in 2016.^5^ Melanoma is largely preventable; more than 80% of newly diagnosed melanomas are associated with an increase in recreational sun exposure and tanning bed use.^6,7^ Melanoma is also largely curable, with 5-year net-standardized survival of 100% for stage-1 tumors, and most tumors among patients with melanoma are stage-1 tumors.^8^ However, among patients presenting with later-stage disease, overall survival rates fall significantly, and once the disease has spread beyond surgical intervention, metastatic melanoma remains largely incurable despite the recent introduction of systemic therapies extending survival.^9,10^

There is growing evidence that time to patient presentation to health care and initial management in primary care are key determinants of patient outcomes for most cancers.^11,12^ Compared with other cancers, melanoma has among the longest delays measured as median time to patient presentation.^13,14^ To understand possible reasons, recent studies have explored symptom appraisal and help-seeking among individuals recently diagnosed with melanoma. Factors contributing to later presentation to health care include having limited awareness of the seriousness of some skin changes, considering changes in skin as part of normal aging, and having concerns about wasting their own and their general practitioners’ time.^15^

Mass media campaigns have been the main population approach to reduce sun exposure and improve time to presentation. In Australia, public health campaigns such as Slip-Slap-Slop have been highly effective in primary prevention, and incidence rates for new melanoma are plateauing.^16^ Mass media approaches in the United Kingdom, such as SunSmart and the Be Clear on Cancer Skin Cancer campaign, have been less effective in reducing sun exposure or promoting consultations to health care. Initiatives in the United States have focused on educational and clinician-led approaches to minimizing exposure among children and young adults with fair skin types to UV radiation.^17^ Routine screening of the general population is not currently recommended internationally, although some countries (eg, the United Kingdom, Australia, New Zealand, Germany, the Netherlands) recommend regular skin checks and/or self-examination for certain subsets of patients with increased risk of melanoma.^18^

More targeted interventions aimed at individuals with increased risk of melanoma could promote earlier presentation to health care. There is a great deal of interest in new technological approaches, such as smartphone applications. People have increasing access to smartphone applications; in 2017, more than 77% of the US and UK populations owned a smartphone.^19^ Smartphone applications can be used to inform prevention and awareness messages, photograph skin lesions, and monitor possible skin changes.^20^ We have demonstrated that it is possible to use a simple patient-completed risk assessment tool in the UK family practice setting to stratify the population into those with population risk and those with increased risk of melanoma.^21^ Individuals with increased risk could receive targeted interventions to promote earlier presentation to health care.

We chose the MySkinPal app as an exemplar for this study, following extensive phase 1 evaluation. Having reviewed the availability of potentially suitable smartphone skin self-monitoring (SSM) applications, also known as skin self-examination (SSE) applications,^20^ we undertook qualitative research, using focus groups and interviews, with individuals with increased risk of melanoma to provide in-depth understanding of consumer views on the usefulness and usability of 2 short-listed applications. MySkinPal was selected for its superior ease of use, including photography, straightforward instructions, and built-in notifications to complete future skin self-monitoring^22^ (eFigure in Supplement 1). We aimed to test the SSM application in a family practice population with increased risk of melanoma and measure its effect on time to consultation and consultation rates for skin changes.

## Methods

The trial protocol has been published^22^ and is available in Supplement 2; therefore, the methods are reported briefly here. This study was approved by the Cambridgeshire and Hertfordshire NHS research ethics committee. This report followed the Consolidated Standards of Reporting Trials (CONSORT) reporting guideline.

### Trial Design and Participants

This individually randomized phase 2 clinical trial was conducted in 12 family practices in Eastern England. Eligible participants were general practice attendees aged 18 to 75 years who owned a smartphone and were identified as having increased risk of melanoma after completing an electronic questionnaire (ie, MelaTools Q risk assessment tool^21^) using tablet computers. Participants were able to read and write English and give informed consent. Exclusion criteria were having a severe psychiatric or cognitive disorder or a physical disorder severe enough to inhibit the use of a smartphone. Potentially eligible patients were given a trial appointment at their general practice. Randomization was performed after written informed consent had been obtained.

### Control

A research nurse invited participants in the control group to complete the baseline questionnaire; the nurse and participants then had a brief discussion about skin health, using the Cancer Research UK leaflets “Be sunsmart—cut your cancer risk” and “Skin cancer—how to spot the signs and symptoms.” Participants then received usual care at their general practice.

### Intervention

In addition to consultation with a nurse, participants randomized to the intervention group had the SSM application loaded on their smartphones and were given verbal and written instructions on its use. A monthly prompt to self-monitor for skin changes was set on the applications, and participants were reimbursed with a voucher for £10 (US $13) to cover the cost of the application.

### Outcomes and Measures

We collected information on sociodemographic and clinical variables as follows: age, sex, marital status, postcode, highest educational level, occupation, history of skin cancer, skin and hair type, and number of raised moles on both arms at baseline.^21,23^ The coprimary clinical outcomes of this trial were as follows: (1) family practice consultation rates and (2) patient interval (ie, time between first noticing a change and consultation^24^) for any skin changes or pigmented skin lesions. Data on consultations in the year before the trial and for 12 months after the trial consultation were collected through audits of general practitioner medical records. Data on the patient interval were measured using the Skin Questionnaire. This self-completed questionnaire, based on the Symptom instrument,^25,26,27^ obtains data on presenting symptoms and their duration before consultation. Monthly electronic searches of general practitioner medical records identified recent skin consultations. Participants were sent (electronically or by mail) a Skin Questionnaire to complete regarding skin changes associated with that consultation.

This was a phase 2 randomized clinical trial of a complex intervention,^28^ so we collected feasibility outcomes, particularly regarding recruitment and retention. Our outcomes were additionally designed to test whether the intervention had the potential to facilitate the early detection of melanoma by evaluating its effect across a range of intermediate end points. The following outcomes were assessed at baseline and in 6-month and 12-month follow-up questionnaires: (1) skin self-examination benefits and barriers, a 10-item scale, validated in the United States among melanoma survivors^29^; (2) self-efficacy for consulting without delay, an 8-item scale adapted from a 10-item scale^30^; (3) perceived melanoma risk, 2 items^29^; and (4) sun protection habits scale, a 5-item measure validated in a US skin cancer prevention program^31^ with additional questions about sun protection. We also assessed potential harms from the intervention in the 6-month and 12-month follow-up questionnaires, as follows: (1) the Melanoma Worry Scale, a 4-item scale with a range of 4 to 17, with higher scores suggesting increased anxiety^32^; (2) the Hospital Anxiety and Depression Scale, a widely used 14-item scale^33^; and (3) quality of life, using the 12-item Short Form Health Survey scale.^34^

Participant-completed measures were collected at baseline, 1 month, and 12 months. Health service utilization data were collected at 12 months by general practice medical record audit.

### Sample Size 

This feasibility trial was designed to have a sample size of 200, with 100 participants in each group. This would provide sufficient data on ease of recruitment and attrition and to estimate effect size to inform a future phase 3 randomized clinical trial.^22^

### Randomization

Eligible patients who consented to participate were randomized 1:1 to either the control or intervention group. Randomization was performed using an online system, and a block randomization method was applied using computer-generated, randomly permuted blocks of sizes 2, 4, and 6, established by a professional independent randomization service (King’s College London Clinical Trials, United Kingdom).

### Masking

Outcomes assessed by self-report obviated the need for researcher masking. For the extraction and analysis of health service data, research staff were masked to group assignment. All statistical analyses were performed masked to group assignment.

### Statistical Analysis

We wrote a statistical analysis plan (agreed on by F.M.W., M.E.B., and C.L.S.), including outlines of all results tables and all analysis code, before exploring any data, and unmasking only occurred after all the analyses had been completed. These methods followed our previously reported approach to this analysis, published in the study protocol. This plan appears in Supplement 2.^22^

Poisson regression was used to analyze the consultation rate primary outcome, and linear regression was used to analyze the patient interval primary outcome, with practice-level random effects to account for possible clustering of outcomes within general practices. Secondary outcomes were analyzed using linear (continuous outcomes) or logistic (binary outcomes) regression, with participant-level random effects to account for the similarity of answers when participants responded at both 6 and 12 months follow-up. Formal statistical comparisons were only made when rates of missing data were similar (prespecified as a difference of 10 percentage points or less). We made no correction for multiple testing, using a 2-sided *P* < .05 as the threshold for statistical significance. All analyses were performed using Stata version 15 (StataCorp).

## Results

We recruited patients from 12 general practices in 7 clinical commissioning groups (CCGs) across eastern England, with a range of list sizes (1 practice [8.3%], <10 000 patients; 8 practices [66.7%], 10 000-20 000 patients; 3 practices [25.0%], >20 000 patients) and locations (3 [25.0%] rural; 1 [8.3%] urban; 8 [66.7%] mixed rural and urban). Between August 22, 2016, and January 6, 2017, we assessed 1729 family practice attendees for eligibility, of whom 1319 (76.3%) did not meet our eligibility criteria (ie, they did not have an increased risk of melanoma), leaving 410 (23.7%) who were eligible. A total of 238 eligible patients (58.0%) consented to be randomized (Figure), with an overall median (interquartile range) age of 55 (43-65) years, 131 (55.0%) women, and 227 (95.4%) white British participants.

**Figure.**
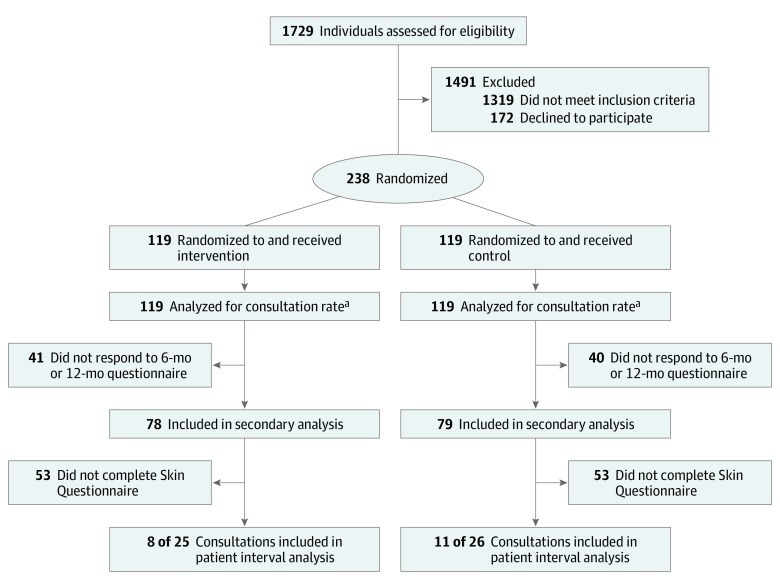
Study Flow Chart ^a^Although 16 participants were lost to follow-up, they were included in the analysis of consultation rate, with the assumption that they did not have consultations.

Table 1 presents baseline data on the trial cohort. The 238 participants (119 randomized to the intervention group and 119 to the control group) were well matched, with no obvious differences in any of the measures collected at baseline. People older than 60 years were less likely to complete the initial assessment compared with people younger than 60 years (236 of 322 [73.3%] vs 152 of 374 [40.6%], *P* < .001). There was no difference in consent rates by sex, and no patients were diagnosed with melanoma during the 12-month follow-up period. A total of 16 participants (6.7%) were lost to follow-up during the trial. Comparing baseline melanoma incidence data during 3 years (2013-2015), provided to the research team by the English National Cancer Registry,^35^ there was no evidence of difference between the study practices and those in the rest of England.

**Table 1.** Baseline Characteristics of Study Participants

Characteristic	Participants, No. (%)		
	All (N = 238)	Control (n = 119)	Intervention (n = 119)
Age, median (IQR), y	55 (43-65)	56 (47-67)	54 (42-62)
Women	131 (55.0)	72 (60.5)	59 (49.6)
Ethnicity			
White British	227 (95.4)	116 (97.5)	111 (93.3)
All other ethnic groups	11 (4.6)	3 (2.5)	8 (6.7)
Education			
None	23 (9.7)	12 (10.1)	11 (9.2)
Secondary school qualifications	109 (45.8)	58 (48.7)	51 (42.9)
University degree	106 (44.5)	49 (41.2)	57 (47.9)
Employment			
Retired	71 (29.8)	38 (31.9)	33 (27.7)
Not in work	21 (8.8)	12 (10.1)	9 (7.6)
Working part-time	50 (21.0)	24 (20.2)	26 (21.8)
Working full-time	96 (40.3)	45 (37.8)	51 (42.9)
Williams melanoma risk score, mean (SD)	31.8 (6.3)	31.7 (5.7)	32.0 (6.8)
Hair color			
Dark brown	41 (17.2)	18 (15.1)	23 (19.3)
Light brown	104 (43.7)	54 (45.4)	50 (42.0)
Blonde	52 (21.8)	26 (21.8)	26 (21.8)
Red	41 (17.2)	21 (17.6)	20 (16.8)
Raised moles			
0	41 (17.2)	22 (18.5)	19 (16.0)
1	39 (16.4)	18 (15.1)	21 (17.6)
2	24 (10.1)	16 (13.4)	8 (6.7)
≥3	134 (56.3)	63 (52.9)	71 (59.7)
Freckles			
None	12 (5.0)	8 (6.7)	4 (3.4)
A few	62 (26.1)	27 (22.7)	35 (29.4)
Several	51 (21.4)	27 (22.7)	24 (20.2)
A lot	113 (47.5)	57 (47.9)	56 (47.1)
Times sunburned in childhood			
0	30 (12.6)	16 (13.4)	14 (11.8)
1-4	106 (44.5)	53 (44.5)	53 (44.5)
5-9	55 (23.1)	28 (23.5)	27 (22.7)
≥10	47 (19.7)	22 (18.5)	25 (21.0)
History of melanoma	12 (5.0)	5 (4.2)	7 (5.9)
History of basal cell carcinoma	26 (10.9)	11 (9.2)	15 (12.6)
History of squamous cell carcinoma	5 (2.1)	4 (3.4)	1 (0.8)

Abbreviation: IQR, interquartile range.

The primary outcome of rate of family practice skin consultation in the year following the intervention was determined for 222 of 238 participants (93.3%). Analysis of consultation rate included all patients and assumed that the 16 participants (6.7%) who were lost to follow-up during the trial had no skin consultations after they were lost to follow-up. The patient interval was determined for 19 of 51 participants (37.3%) who had skin consultations; 8 of 25 (32.0%) in the intervention group and 11 of 26 (42.3%) in the control group completed a Skin Questionnaire after a relevant family practice consultation. Secondary outcomes were available for 157 participants (66.0%) for at least 1 point.

### Coprimary Outcomes

There was no evidence of difference in consultation numbers and rates between the control group and the intervention group (adjusted risk ratio, 0.96; 95% CI, 0.56-1.66; *P* = .89). However, the mean (SD) consultation rate per person in the intervention group was higher in the 12 months before the trial than in the 12 months during the trial (0.29 [0.61] vs 0.21 [0.66]) (eTable in Supplement 1). Adjusting for age, sex, and clustering within general practices did not change the results. We did not perform formal statistical testing for the patient interval coprimary outcome because of differences in the rates of missing data between the control and intervention groups (Table 2).

**Table 2.** Comparative Results for Primary and Secondary Outcomes Across Follow-up

Outcome	Comparison Type^a^	Estimate (95% CI)	*P* Value
Coprimary outcomes			
Consultation rate per person per year	Adjusted risk ratio	0.96 (0.56 to 1.66)	.89
Patient interval, d	Adjusted mean difference	−20.2 (−90.4 to 50.0)	NA^b^
Secondary outcomes with validated scales			
Skin self-examination benefits score	Adjusted mean difference	0.08 (−0.83 to 1.00)	.86
Skin self-examination barriers score	Adjusted mean difference	−0.29 (−1.83 to 1.25)	.71
Self-efficacy for consulting without delay	Adjusted mean difference	0.20 (−3.69 to 4.10)	.92
Perceived risk of getting melanoma “higher” or “much higher” than other people	Descriptive results only^c^	NA	NA
Perceived lifetime risk of melanoma	Descriptive results only^c^	NA	NA
Sun protection habits score	Adjusted mean difference	0.12 (−0.01 to 0.24)	.07
Additional secondary outcomes			
“Often” or “always” practiced sun protection in the past year	Descriptive results only^c^	NA	NA
“Extremely likely” or “likely” to practice sun protection in the coming year	Descriptive results only^c^	NA	NA
Sunburned at least once in the last year	Descriptive results only^c^	NA	NA
Measures of possible harm			
Melanoma Worry Scale	Adjusted mean difference	−0.12 (−0.56 to 0.31)	.58
Short Form Health Survey, physical component summary	Adjusted mean difference	−0.31 (−2.39 to 1.76)	.77
Short Form Health Survey, mental component summary	Adjusted mean difference	1.28 (−0.34 to 2.90)	.12
HADS Depression score	Adjusted mean difference	−0.43 (−1.19 to 0.33)	.26
HADS Anxiety score	Adjusted mean difference	0.11 (−0.67 to 0.90)	.78

Abbreviations: HADS, Hospital Anxiety and Depression Scale; NA, not applicable.

^a^ All comparisons were intervention vs control.

^b^ No formal statistical testing for the patient interval outcome was performed because of differences in the rates of missing data between the control and intervention groups.

^c^ Because of small numbers of participants who provided data for this outcome, only descriptive results were calculated.

### Secondary Outcomes

Table 2, Table 3, and Table 4 present the results of the participant-reported secondary outcomes, ie, skin self-examination benefits and barriers, self-efficacy for consulting without delay, perceived melanoma risk, sun protection habits, melanoma worry, anxiety and depression, and quality of life. There were no statistically significant differences between trial groups on any of the secondary outcome measures (eg, measures of SSM: adjusted mean difference, 0.08; 95% CI, −0.83 to 1.00; *P* = .86; Melanoma Worry Scale: adjusted mean difference, −0.12; 95% CI, −0.56 to 0.31; *P* = .58). A sensitivity analysis additionally adjusting for age and sex showed similar results.

**Table 3.** Descriptive Results of Continuous Secondary Outcomes at 6-Month and 12-Month Follow-up

Secondary Outcome	Scale		Follow-up, mo	Control Group (n = 119)		Intervention Group (n = 119)	
	Range	Interpretation		Respondents, No. (%)	Mean (SD)	Respondents, No. (%)	Mean (SD)
**Measures of potential benefit**							
Skin self-examination benefits score	7-35	Higher score indicates greater benefit	6	56 (47.1)	26.89 (3.81)	56 (47.1)	27.52 (4.04)
			12	65 (54.6)	27.43 (3.64)	64 (53.8)	27.67 (3.47)
Skin self-examination barriers score	10-50	Higher score indicates more barriers	6	56 (47.1)	26.61 (5.74)	56 (47.1)	25.43 (6.28)
			12	65 (54.6)	25.69 (6.66)	64 (53.8)	25.03 (5.74)
Self-efficacy for consulting without delay	8-80	Higher score indicates less delay	6	56 (47.1)	55.95 (17.76)	56 (47.1)	57.25 (14.43)
			12	65 (54.6)	57.17 (17.37)	64 (53.8)	57.06 (16.80)
Perceived lifetime risk of melanoma	0%-100%	Higher percentage indicates higher perceived risk	6	56 (47.1)	47.7 (21.4)	56 (47.1)	49.1 (24.4)
			12	65 (54.6)	45.6 (22.8)	64 (53.8)	48.4 (23.9)
Sun protection habits score	1-4	Higher score indicates better habits good	6	56 (47.1)	2.43 (0.64)	56 (47.1)	2.57 (0.48)
			12	65 (54.6)	2.48 (0.59)	64 (53.8)	2.57 (0.51)
**Measures of possible harms**							
Melanoma Worry Scale	4-17	Higher score indicates more worry	6	56 (47.1)	6.70 (2.20)	56 (47.1)	6.84 (1.90)
			12	65 (54.6)	6.37 (1.66)	64 (53.8)	6.52 (1.83)
Short Form Health Survey 12, physical component summary	0-100	Higher score indicates better physical health	6	56 (47.1)	49.10 (10.85)	56 (47.1)	48.82 (11.43)
			12	65 (54.6)	48.94 (11.36)	64 (53.8)	49.91 (10.82)
Short Form Health Survey 12, mental component summary	0-100	Higher score indicates better mental health	6	56 (47.1)	41.29 (6.69)	56 (47.1)	41.67 (5.46)
			12	65 (54.6)	40.27 (7.42)	64 (53.8)	42.54 (4.77)
HADS depression score	0-21	Higher score indicates worse depression symptoms	6	56 (47.1)	4.05 (3.60)	56 (47.1)	3.27 (3.27)
			12	65 (54.6)	4.08 (4.24)	64 (53.8)	2.47 (2.33)
HADS anxiety score	0-21	Higher score indicates worse anxiety symptoms	6	56 (47.1)	5.86 (4.00)	56 (47.1)	5.71 (4.20)
			12	65 (54.6)	5.83 (4.20)	64 (53.8)	5.34 (3.96)

Abbreviation: HADS, Hospital Anxiety and Depression Scale.

**Table 4.** Descriptive Results on Binary Secondary Outcomes at 6-Month and 12-Month Follow-up

Measure of Possible Harm	Follow-up, mo	No./Total No. (%)	
		Control Group (n = 119)	Intervention Group (n = 119)
Perceived risk of getting melanoma “higher” or “much higher” than other people	6	25/56 (44.6)	28/56 (50.0)
	12	26/65 (50.0)	26/64 (40.6)
“Often” or “always” practiced sun protection in the past year	6	36/56 (64.3)	43/56 (76.8)
	12	47/65 (72.3)	49/64 (76.6)
“Extremely likely” or “likely” to practice sun protection in the coming year	6	47/56 (83.9)	52/56 (92.9)
	12	60/65 (92.3)	59/64 (92.2)
Sunburned at least once in the last year	6	21/56 (37.5)	21/56 (37.5)
	12	15/65 (23.1)	22/64 (34.4)

## Discussion

### Summary

To our knowledge, this is among the first trials worldwide to investigate the potential effect of a behavioral intervention using an SSM application to promote help-seeking in patients with increased risk of melanoma. We combined evidence-based approaches to identify people at increased risk in the family practice setting with new approaches using smartphone applications for SSM. We were unable to demonstrate any effect on consultation rates or patient interval, but importantly, there was no evidence of psychological harm from the intervention during the following 12 months. At the same time, there was also no evidence of any beneficial effect on sun protection habits, skin self-examination behavior, or perceived risk of developing melanoma among those receiving the intervention. Trial recruitment, retention, and initial delivery of the intervention were all feasible. However, we have no data on the actual use of the SSM app; this process measure is an important further dimension to assess before making a feasibility decision regarding a larger trial powered to demonstrate effects on either consultation rates or the primary care interval.

The intervention, including uploading the app, demonstrating use of the app, and providing regular reminders, was not found to alter consultation rates or help-seeking for skin symptoms. This may have been because of the short follow-up time and possibly influenced by seasonal variation in detecting skin changes.^36^ We also found no differences in our quantitative measurements of sun protection habits, skin self-examination, self-efficacy to consult, melanoma risk perception, or measures of general or cancer-specific anxiety.

It is not easy to interpret these findings in the context of other similar studies given that we have not been able to identify any trial evidence using a smartphone application to support SSM or SSE for individuals with increased melanoma risk. There is US trial evidence for the efficacy of SSE skills training and reminders among patients recently diagnosed with melanoma^37^ and among skin clinic patients without melanoma.^38^ Furthermore, the Check It Out randomized clinical trial, set in the United States among a similar population with increased risk of melanoma, reported that increasing SSE practices in the short-term resulted in more abnormal lesions detected and more skin surgical procedures,^39^ although, like our trial, it was not powered to detect melanoma diagnosis. In the United States, most studies have been set among people previously treated for a melanoma and, therefore, with very high risk for development of a new primary melanoma as well as recurrence. These studies have not demonstrated strong evidence that SSE influences melanoma mortality,^40^ although it has been shown to reduce the incidence of thick melanomas.^41^ Other studies set in the United Kingdom and elsewhere have shown that SSE practice is frequently suboptimal,^42,43^ and barriers to initiating and maintaining SSE include lack of initial training, declining motivation, and insufficient time.^44^

These barriers could be addressed by several of the features of smartphone applications that enable self-monitoring among individuals with increased risk of melanoma. Our findings relate to the concept of using an SSM application rather than the properties of the specific application selected following extensive evaluation of more than 40 available applications in 2016.^20^ The Achieving Self-directed Integrated Cancer Aftercare intervention is a rigorously developed, theoretically based, digitally supported application that uses specified behavior-change techniques to prompt users to perform regular, high-quality SSE and gives appropriate clinical responses when they raise a concern^45^; it is currently under evaluation in a UK randomized clinical trial among individuals recently treated for primary melanoma.^22^ Both the Achieving Self-directed Integrated Cancer Aftercare trial and our trial depend on patients using the applications optimally. We have no knowledge of how much the application was used in this trial, although no participants in either trial group reported that they had contaminated the trial by downloading other SSM smartphone applications.

### Limitations

This study has limitations. The study was designed as a phase 2 trial of a complex intervention.^28^ We based the coprimary outcomes of family practice skin consultation rates and the patient interval on the intervention logic model. Encouraging people with increased risk of melanoma to self-monitor their own skin and consult their general practitioner if they have any concerns could reduce potential diagnostic delay for melanoma and could result in the detection of earlier-stage disease. Therefore, the coprimary outcomes were the most suitable intermediate measures along this causal pathway. Nevertheless, no participants were diagnosed with melanoma, and few consulted with their general practitioners during the 12-month follow-up period despite the participant population having increased risk of melanoma. This trial was not designed to have sufficient statistical power to detect a meaningful difference; a much larger trial would be needed for sufficient power to detect differences in the patient interval or skin consultation rates and an even larger trial to detect differences in time to diagnosis, stage at diagnosis, or mortality from melanoma.

Although the feasibility outcomes around recruitment were satisfactory, older individuals were less likely to consent to participate. More than half of those found to be eligible for the trial consented to participate. The randomized groups were well-balanced, and few participants were lost to follow-up over 12 months (16 [6.7%]). However, there were fewer than expected events across both groups (ie, 51 skin consultations) and a low response (ie, 37%) to the Skin Questionnaire, meaning that we were unable to report on the effect of the intervention on the time to consultation (ie, patient interval). The control group received a brief discussion about skin health and Cancer Research UK leaflets. While intended as an attention control, this could potentially have increased consultations about skin changes and reduced the effect size of the intervention. We were unable to accurately assess the use of the application by participants (either by direct or electronic questioning) and cannot say whether it was used regularly for skin self-monitoring or whether that contributed to the absence of effect on consultation rates in the intervention arm. We consider the findings generalizable beyond the East of England because of the recruitment of patients from 12 general practices located in urban and rural areas with a range of size of patient lists and socioeconomic deprivation.

## Conclusions

In this phase 2 randomized trial, the use of a SSM smartphone application did not have an effect on the rate at which participants with higher risk of melanoma consulted with family practice physicians regarding skin changes. Therefore, significant uncertainty remains regarding the best strategies to promote early detection of melanoma. For those with moderate risk of melanoma, behavioral approaches, such as using an SSM smartphone application to prompt timely consultation, remain an option, promoted particularly among policy makers, but our trial does not offer even preliminary randomized clinical trial evidence to support this. While such applications have not been shown to have value in diagnosing melanoma,^46^ they may have more promise as monitoring tools to identify skin changes. Further evidence on how to ensure people with increased risk of melanoma engage with and use these applications and whether this leads to earlier detection is needed. Until then, the role of smartphone applications as a strategy to detect melanoma earlier remains unclear.

## Notes

Supplement 1. **eFigure. **Smartphone Application Screenshots **eTable. **Descriptive Results on Coprimary Outcomes During Trial Follow-up

Supplement 2. **Trial Protocol and Statistical Analysis Plan**

Supplement 3. **Data Sharing Statement**
