# Supplementary material for: Effect of a Skin Self-monitoring Smartphone Application on Time to Physician Consultation Among Patients With Possible Melanoma: A Phase 2 Randomized Clinical Trial
Source: JAMA Netw Open. 2020 Feb 26;3(2):e200001. doi: 10.1001/jamanetworkopen.2020.0001 (PMC7137684; doi:10.1001/jamanetworkopen.2020.0001)
Supplement: Supplement 1. — eFigure. Smartphone Application Screenshots eTable. Descriptive Results on Coprimary Outcomes During Trial Follow-up [file jamanetwopen-3-e200001-s001.pdf]

Supplementary Online Content

Walter FM, Pannebakker MM, Barclay ME, et al. Effect of a skin self-monitoring smartphone application on time to physician consultation among patients with possible melanoma: a phase 2 randomized clinical trial. *JAMA Netw Open*. 2020;3(2):e200001. doi:10.1001/jamanetworkopen.2020.0001

eFigure. Smartphone Application Screenshots

eTable. Descriptive Results on Coprimary Outcomes During Trial Follow-up

This supplementary material has been provided by the authors to give readers additional information about their work.

© 2020 Walter FM et al. *JAMA Network Open.*

eFigure. Smartphone Application Screenshots

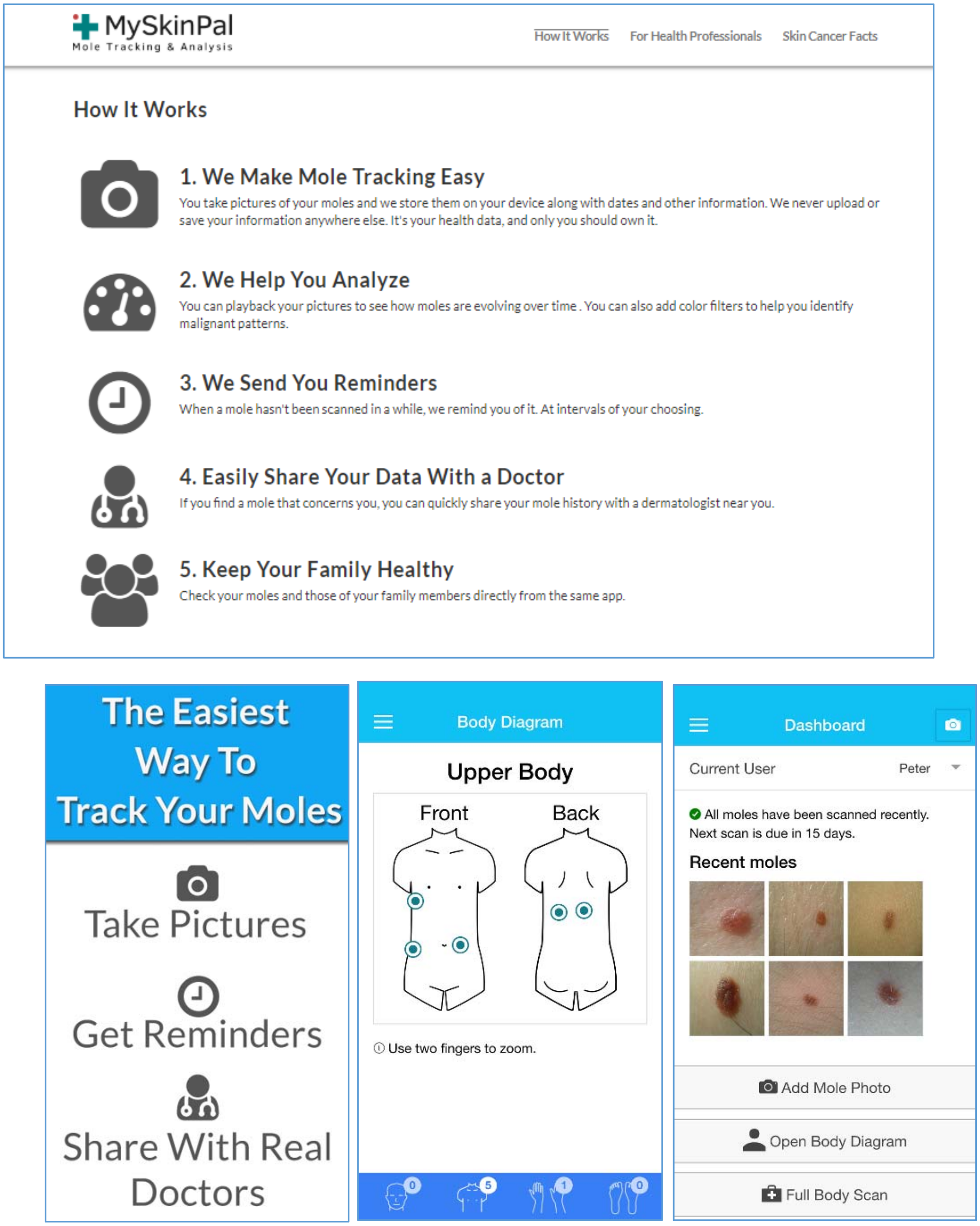

© 2020 Walter FM et al. *JAMA Network Open.*

eTable. Descriptive Results on Coprimary Outcomes During Trial Follow-up

Co-primary outcome	Control N	Control mean	(Control SD)	Intervention N	Intervention mean	(Intervention SD)
Consultation rate per person per year	119	0.22	(0.75)	119	0.21	(0.66)
Patient interval (days)	11	61.1	(96.1)	8	40.9	(35.0)

© 2020 Walter FM et al. *JAMA Network Open.*
