# Supplementary material for: Effect of a Skin Self-monitoring Smartphone Application on Time to Physician Consultation Among Patients With Possible Melanoma: A Phase 2 Randomized Clinical Trial
Source: JAMA Netw Open. 2020 Feb 26;3(2):e200001. doi: 10.1001/jamanetworkopen.2020.0001 (PMC7137684; doi:10.1001/jamanetworkopen.2020.0001)
Supplement: Supplement 2. — Trial Protocol and Statistical Analysis Plan [file jamanetwopen-3-e200001-s002.pdf]

MelaTools SSM Trial

Skin Self-Monitoring for primary care patients at higher risk of melanoma: a phase II RCT

Protocol

Funder: NIHR Clinician Scientist Award NIHR-CS-012-030
Study duration: End of March 2018
Start date: 1st July 2016
Organisation administering award: University of Cambridge

Lead Investigator:
Dr Fiona M Walter, University of Cambridge, UK & University of Melbourne, Australia

Co-Investigators:
Prof Jon Emery, University of Melbourne, Australia & University of Cambridge, UK
Dr Peter Murchie, University of Aberdeen, UK

Collaborators:
Dr Nigel Burrows, Cambridge University Hospitals NHS Foundation Trust, UK
Mr Per Hall, Cambridge University Hospitals NHS Foundation Trust, UK
Dr Kate Williams, Senior Trial Manager / Senior Research Associate, Primary Care Unit, University of Cambridge, UK
Dr Katherine Saunders, Senior Statistician, Primary Care Unit, University of Cambridge, UK

Study Co-ordinator:
Dr Katie Mills, University of Cambridge, UK

Research Assistant:
Rebecca Lantaff, University of Cambridge, UK

Table of Contents

1. Abbreviations
2. Protocol Synopsis
3. Funding
4. Co-Investigators and Collaborators
   4.1 Patient and Public Involvement and Engagement (PPIE)
5. Study Schema
6. Aims and Hypotheses
   6.1 Purpose
   6.2 Aims
   6.3 Hypotheses
7. Background
   7.1 Melanoma
   7.2 Patient Interval
   7.3 Smartphone Applications
   7.4 Skin Self-Monitoring
   7.5 Phase I studies
      7.5.1 MelaTools Q study
      7.5.2 MelaTools-Apps study
8. Research Methods
   8.1 Study Design and Outcomes
   8.2 Study setting
   8.3 Intervention
      8.3.1 Development of standardised consultation and intervention
      8.3.2 Training for intervention delivery
      8.3.3 Fidelity of intervention delivery
   8.4 Recruitment procedure and Participant eligibility/exclusion criteria
   8.5 Randomisation
   8.6 Trial consultation
      8.6.1 Control and Intervention groups
      8.6.2 Intervention group only
   8.7 Follow up
      8.7.1 Participants
      8.7.2 Primary care
      8.7.3 Cancer Registry
   8.8 Measures
      8.8.1 Patient reported outcome measures (PROMs)
      8.8.2 Other measures
      8.8.3 Follow up
   8.9 Reimbursement
   8.10 Withdrawals and protocol deviations
9. Data management
   9.1 Participant data
   9.2 Outcome Registry Intervention and Operation Network (ORION)
   9.3 Study management
   9.4 Sample size and power calculation
   9.5 Data analysis
10. Outputs
11. References

1. Abbreviations

CRN	Comprehensive Research Network
CRUK	Cancer Research United Kingdom
GP	General Practitioner
HADS	Hospital Anxiety and Depression Scale
NIHR	National Institute for Health Research
ORION	Outcome Registry Intervention and Operation Network
PI	Principal Investigator
PM	Practice Manager
SSM	Skin self-monitoring
RCT	Randomised Controlled Trial
RISP	Research Information Sheet for Practices
SMS	Short messaging service

2. Protocol Synopsis

Title	Skin-Self Monitoring for primary care patients at higher risk of melanoma: a phase II RCT
Short Title	MelaTools SSM Trial
Funding	NIHR- Clinician Scientist Award
Chief Investigator	Dr Fiona Walter
Research Purpose	To test a novel skin self-monitoring (SSM) intervention in general practice aimed at promoting timely consulting by people at 'above-population' risk of melanoma
Aims	1. To evaluate SSM App use compared with standard information on skin change appraisal and help-seeking among patients at 'above-population' risk of melanoma;<br>2. To optimise the intervention, to establish its acceptability and to collect all the relevant data to inform a subsequent phase III trial.
Study Design	Phase II, multi-site RCT
Study setting	Patients with an 'above-population' risk of melanoma will be recruited from general practices in CRN Eastern.
Sample size & recruitment	Based on our recent MelaTools Q study, we anticipate that we will need to approach approximately 2,000 people to have 1,600 completed questionnaires; 400 people (25%) will be at higher risk and eligible, and 50% of these will need to attend the trial consultation in order to reach our target of 200 participants.
Inclusion Criteria	• ≥18 years of age<br>• ≤ 75 years of age<br>• Ability to read and write for informed consent and smartphone use
Exclusion Criteria	• Severe psychiatric or cognitive disorder<br>• Physical disorder severe enough to inhibit the use of a smartphone<br>• No smartphone
Randomisation	Patients will be randomised 1:1 to either control or intervention group.
Intervention	All participants in both groups will have a standardised consultation about prevention of skin cancer with the research nurse. Those in the intervention group will also be provided with a SSM App and standardised tuition on use.
Duration of individual's participation	Study participation will last no longer than 12 months; patient-reported outcomes will be collected at baseline, 6 months and 12 months post-consultation.
Process and Outcome Measures	We will examine processes relating to use of the SSM App, including:<br>• Patient outcomes: patient interval (time from first noticing skin change to consultation); sun protection habits, skin self-examination, melanoma worry and perceived melanoma risk, self-efficacy for consulting without delay; anxiety, depression and quality of life;<br>• Clinical outcomes: melanoma (and other skin cancer) diagnosis; actions during consultations: diagnosis, referral, excision, monitor;<br>• Practice outcomes: consultation rates;<br>• Trial processes: feasibility and acceptability;<br>• Incidence of melanoma (and other skin cancer) diagnosis across participating practices and their regions during the trial and after 5 years.
Study duration	It is anticipated that the study will be completed by March 2018.

3. Funding

Dr Fiona Walter received a NIHR Clinical Scientist award (NIHR-CS-012-030) in June 2012 to fund the MelaTools programme. This is the final study in this programme.

4. Co-Investigators and Collaborators

Principal Investigator	University	Country	Role
Dr Fiona Walter	University of Cambridge	England	Management and oversight of the study
**Co-Investigators**			
Prof Jon Emery	University of Melbourne	Australia	Advising on the design, implementation and analysis of the study
Dr Nigel Burrows	Cambridge University Hospitals NHS Foundation Trust	England	Advising on the design, implementation and analysis of the study
Mr Per Hall	Cambridge University Hospitals NHS Foundation Trust	England	Advising on the design, implementation and analysis of the study
Dr Peter Murchie	University of Aberdeen	Scotland	Advising on the design, implementation and analysis of the study
Dr Katie Mills	University of Cambridge	England	Co-ordinating and conducting the research
**Collaborators**			
Dr Juliet Usher-Smith	University of Cambridge	England	Advising on the design, implementation and analysis of the study
Or Katherine (Katie) Saunders	University of Cambridge	England	Statistical support
Dr Kate Williams	University of Cambridge	England	Liaison with Cambridge Clinical Trials Unit, expertise in trial management

4.1 Patient and public involvement and engagement (PPIE)

Three experienced PPIE representatives have provided their input on the study design and materials, and will contribute to the overall interpretation of the study findings, and dissemination of the study results:

- Mrs Patricia Fairbrother, member of the NCRI Skin Cancer Clinical Studies Group
- Mr Simon Rodwell, Chief Executive, Melanoma Focus & member of the NCRI Skin Cancer Clinical Studies Group
- Mrs Margaret Johnson, member of the NCRI Primary Care Clinical Studies Group: Early Diagnosis sub-group & lay representative on the Primary Care Steering Group: CRN Eastern

5. Study Schema

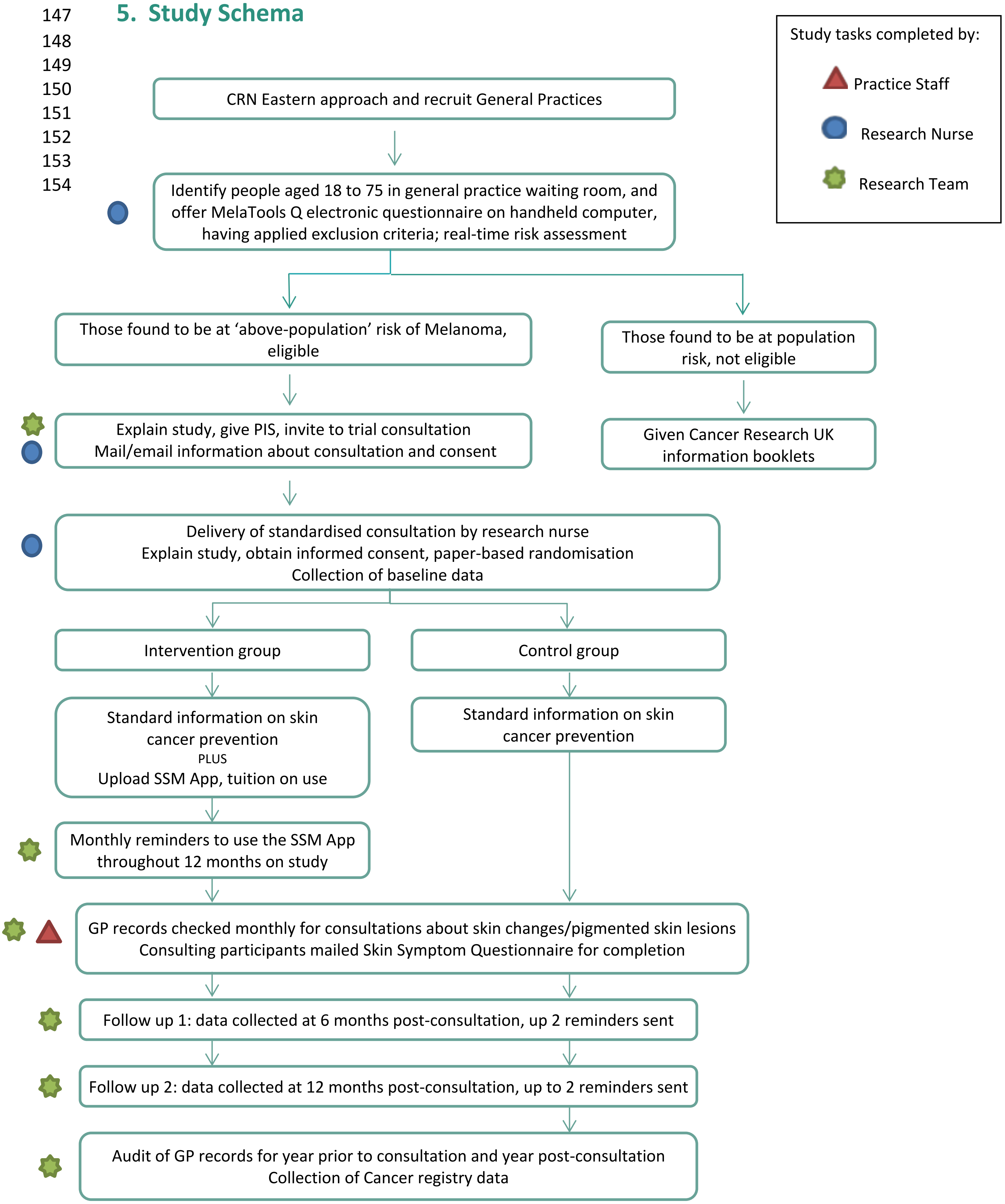

6. Aims and Hypotheses

6.1 Purpose

This phase II pilot randomised controlled trial (RCT) tests a novel intervention of the use of a Skin Self-Monitoring (SSM) smartphone application ('App'), and self-monitoring monthly reminders, to promote timely consulting for skin changes/pigmented skin lesions by people at above-population risk of melanoma.

6.2 Aims

1. To assess the effect of using a SSM App compared with standard information on the patient interval among patients at above-population risk of melanoma;
2. To obtain preliminary estimates of effect across a range of outcome measures to inform selection of the primary outcome for a definitive phase III trial.

6.3 Hypotheses

1. The MelaTools SSM Trial intervention will increase the number of consultations for pigmented skin lesions in people at above-population risk of melanoma;
2. The MelaTools SSM Trial intervention will be acceptable and will reduce the patient interval (including symptom appraisal and help-seeking intervals) in people at above-population risk of melanoma;
3. The MelaTools SSM Trial intervention will not cause significant distress or worry in people at above population risk of melanoma.

7. Background

7.1 Cutaneous malignant melanoma

Melanoma is the leading cause of skin cancer deaths in the UK with 2,148 in 2012 (1). Melanoma incidence has quadrupled over the last 30 years and is continuing to rise (2). Although it is more common with increasing age, it is disproportionately high in younger people (3, 4). Risk factors include fair skin, family history of melanoma, multiple naevi, and sun damage. Melanoma is associated with significant morbidity, and the thickness of the lesion at diagnosis is the most important prognostic factor: stage 1 disease has 5-year survival rates of over 95%, compared to less than 40% for stage 4 disease (5). The UK has lower 1- and 5-year melanoma survival rates than comparable countries in Europe (6). Diagnostic delays are thought to contribute to this, and there is evidence of avoidable delay; the UK policy focus on identifying and reducing these delays has aimed to save 5-10,000 lives a year across all cancers (7). Although findings from the SCREEN project in Germany suggests that population screening may have a substantial impact on melanoma incidence and 5 year mortality (8), routine screening of the general population using a total body skin examination is not currently recommended in the UK. Therefore a focus on secondary prevention through early, timely detection and prompt treatment could make an important contribution to melanoma outcomes.

7.2 Patient Interval

Current literature demonstrates that patient pathways to presentation and management in primary care are key determinants in cancer outcomes (9, 10). When compared with people diagnosed with other cancers, those diagnosed with melanoma have the second longest median time between first noticing a symptom and presenting to primary care (patient interval) (11). There is a need to optimise this time period by assessing the factors which influence the patient interval, namely symptom appraisal and help-seeking intervals (12), for skin changes suspicious of melanoma. Our team's studies have highlighted the need to provide patients with clear information on the

symptoms of melanoma and guidance on monitoring skin changes (13, 14). Policy and third sector approaches have aimed to raise symptom awareness and promote timely help-seeking. More targeted approaches could prioritise education for those patients at higher risk, and promote tools and new technologies that focus on detecting early signs of melanoma.

7.3 Smartphone Applications

Smartphones have been termed 'the new clinical tools in oncology' (15). Most (>75%) of the UK population now own a smartphone, and they have quickly changed from being devices for communication to include specialized applications ('Apps') that are intimately involved in many aspects of daily life. Many Apps now focus on health issues, presenting new opportunities for risk assessment, symptom appraisal, monitoring symptoms and signs over time, and cues to seek professional advice. The use of such Apps for detecting skin changes could potentially assist in earlier diagnosis of melanoma, in prompting users to monitor their pigmented skin lesions over time, and in suggesting professional, timely review of any suspicious moles detected. Before healthcare providers can recommend an App to their patients they need to be confident that the App will not cause any harm, will be user-friendly, and will aid recognition of the target disease or behaviour (16).

7.4 Skin Self-Monitoring (SSM)

Up to 75% of melanomas are detected by people or their family/friends (rather than health care professionals) (17). To maximise the effectiveness of SSM the person performing the examination should be able to identify skin changes and features of skin lesions which could indicate melanoma, yet recent studies have demonstrated that suspicious signs of early skin changes may not be widely known (18,14). Educating patients and the public is possible: newly diagnosed Italian melanoma patients who performed self-skin examination were found to have thinner tumours (19), and Australian melanoma patients were found to adhere to medical advice on skin self-examination during follow-up care (20). A recent Scottish study has demonstrated melanoma patients are prepared to use digital technology to support them in conducting SSM during follow-up (21). We therefore set out to explore using mobile technology for SSM among people at higher risk of melanoma in the primary care setting to encourage timely consultation for possible melanoma.

7.5 Phase I studies

Two recent studies have provided data to inform the Phase II RCT.

7.5.1 MelaTools Q Study

Defining higher-risk populations using risk prediction models may help targeted screening and early detection approaches. We recruited participants from the waiting rooms of 22 general practices covering a total population of >240,000 in three UK regions: Eastern England, Northeast Scotland, and North Wales. Participants completed an electronic questionnaire incorporating the Williams melanoma risk model (22) using tablet computers. 7,742/9,004 approached people completed the electronic questionnaire (86%). The mean melanoma risk score for the 7,566 eligible participants was 17.15 (SD 8.51), with small regional differences, mainly due to greater freckling and childhood sunburn among Scottish and Welsh participants. We concluded that collecting data on the melanoma risk profile of the general population in UK primary care is both feasible and acceptable, and provides opportunities for new methods of real-time risk assessment and risk stratified cancer interventions (23).

7.5.2 MelaTools-Apps Study

This recent study aimed to understand community user views on the usefulness and usability of Apps for SSM. Participants were recruited through general practice waiting rooms via poster advertisements, and stratified into two melanoma risk groups using the MelaTools Q risk assessment questionnaire. Those at above-population risk were invited to participate in an introductory workshop, a diary to complete over 3 months, and then a face to face interview. Each participant

tested one or more of the following Apps: FotoSkin, MoleMonitor, SkinVision, UMSkinCheck and MySkinPal. All these Apps encourage photographing skin changes, and suggest that the user compares these images over time. Other options include completion of a full body check with prompts to monitor certain body areas offered, and skin cancer awareness information. The initial results from this study have provided insight into the usability of these Apps, and guided decision-making on which App to be used in this phase II trial.

8. Research Methods

8.1 Study Design and Outcomes

We applied the well-established MRC methodological framework for the design and evaluation of complex interventions to provide preliminary data to inform the design of this phase II RCT (24, 25). As this is a feasibility trial there will be two primary outcomes:

Primary Outcome/s

a. *Consultation rates* for any skin changes/pigmented skin lesions presented to their GP/practice nurse during the 12 months following the trial consultation compared with the 12 months prior to the trial and;

b. *The patient interval (PI)* for all skin changes/pigmented skin lesions presented to their GP/practice nurse during the 12 months following the trial consultation.

Secondary Outcomes will include:

a. *Patient-Reported Outcomes,* including: sun protection habits, skin self-examination, melanoma worry and perceived melanoma risk, self-efficacy for consulting without delay; anxiety, depression and quality of life;

b. *Trial feasibility and acceptability*, including: data on patient recruitment, attrition, and response rates to outcome measures to inform decisions about a future phase III trial;

c. *Melanoma incidence* across participating practices, to contextualise trial findings and after 5 years.

8.2 Study setting and practice recruitment

Participants will be recruited via general practices in the East of England. GP practice recruitment will be supported by CRN:Eastern who will approach practices in the designated geographical regions, favouring Research Site Initiative (RSI) practices. Recruitment will focus on RSI practices because these practices have an ongoing commitment to research and, generally, have allocated research nurse time. In practices which do not have funded research nurse time, a research nurse from the CRN will be funded to support the study.

8.3 Intervention

8.3.1. Development of standardised consultation and intervention

A detailed consultation script and training module developed by the research team will guide the research nurses conducting the consultation and delivering the intervention. The instructions for using the SSM App have been piloted in the MelaTools-Apps study.

8.3.2 Training for intervention delivery

The research team (FW, KM & BL) will deliver an interactive training session to CRN:Eastern research nurses, detailing the delivery of the control and intervention components of the trial consultation. This will include a PowerPoint presentation of the study design, and instructions on the delivery of the consultation. The instructions will detail the standardised information for all participants on skin cancer prevention, based on Cancer Research UK's publications 'Skin Cancer: How to spot the signs and symptoms', and 'Be sun-smart: cut your cancer risk'. The intervention participants will also have the SSM App installed onto their smartphone, with instructions on its use. A manual and

consultation script will be provided, and the nurses will have time to familiarise themselves with the SSM App. There will be the opportunity to practice the consultation and receive feedback.

8.3.3 Fidelity of intervention delivery

To ensure fidelity of the intervention delivery, the study co-ordinator (KM) will observe and record each of the nurses conducting study consultations. A 10% sample of consultations will be observed during the trial period. Observations will be completed on a 2 monthly basis during the recruitment period to monitor and review the use of the consultation scripts and delivery of the intervention and control group consultations. A simple checklist will be used to score each observed consultation, in line with previous research assessing fidelity in a primary care based intervention (26).

8.4 Recruitment Approach & Participant eligibility/exclusion criteria

The recruitment approach developed for the MelaTools Q Study was very successful (7,742/9,004 approached people completed the electronic questionnaire- 86%); the same recruitment strategy will be used in this trial. People aged between 18-75, attending the general practice as a patient or companion, will be invited to take part. Researchers will recruit participants opportunistically in the reception area at different times of the day and different days of the week, in order to ensure a broad range of ages, gender, and educational level are approached. People will not be invited to take part if they exhibit any of the exclusion criteria:

- Severe psychiatric or cognitive disorder;
- Inability to read English to a reasonable standard;
- Physical disorder severe enough to inhibit the use of a smartphone.

Those willing to take part will be invited to complete an electronic questionnaire using tablet computers. The gender and reason for not wishing to participate will be recorded for each person choosing not to take part.

The electronic questionnaire's MelaTools Q risk assessment tool will be preceded by a consent form. Real-time risk assessment will then stratify respondents into 'population risk' (majority) and 'above-population risk' (minority) groups. People at 'population risk' will be thanked, and given the Cancer Research UK leaflets on risk factors of melanoma and melanoma prevention advice. People at 'above-population' risk will be invited to participate in the trial, and given the Participant Information Sheet and an appointment time within 14 days for the SSM Trial consultation. This will be confirmed by email or post as the potential participant prefers.

8.5 Randomisation

At the trial consultation, and once fully consented, participants will be randomised 1:1 to either the control arm (standard information) or to the intervention arm (standard information and SSM App). A block randomisation method, using computer-generated, randomly permuted blocks of size 2, 4, and 6, established by the trial statistician, will be applied. Sets of numbered, sealed envelopes will be prepared, with the order of the sequences verified on completion of the trial.

8.6 Trial Consultation

8.6.1 Control and Intervention groups

The research nurse will start all the trial consultations by taking written informed consent. Participants will then complete the baseline questionnaire on an iPad. Next, the research nurse will deliver the standardised advice on skin cancer prevention, and provide written or emailed supporting information sheets. The follow-up procedure will be explained (when they will receive questionnaires, how long they have to complete them, how they can receive help answering questions etc.). Participants who fail to attend the consultation will be contacted for another appointment.

8.6.2 Intervention group only

The research nurse will help intervention group participants to download the SSM App onto their smartphone; they will go on to give instructions on its use, again supported by written or emailed information sheets. Finally, each intervention participant will be given an Apple Apps store/Google Play voucher to pay for the App. A monthly SMS will be sent to these participants to prompt them to think about symptoms and to use the SSM App.

8.7 Follow-up

8.7.1 Participants

Six months post-study consultation, all participants will be sent an email reminding them of the study, and a link to an online questionnaire. The questionnaire will be identical to the baseline questionnaire (omitting the demographic data), and will take up to 15 minutes to complete. The same will happen 6 months later, at 12 months post consultation. If either the 6 and/or 12-month follow-up questionnaire have not been returned after 2 weeks, a further reminder will be sent; this will be repeated after a further 2 weeks. On return of the questionnaire the HADS will be scored; if the participant scores ≥10 on the anxiety scale or ≥8 on the depression scale, a letter will be sent to notify their GP.

8.7.2 Primary care

The GP practice managers will run a monthly search of their general practice's electronic medical records to identify all study participant consultations with GPs and practice nurses. If a consultation concerned a skin change/pigmented skin lesion, a Skin Symptom Questionnaire will be sent to the participant, with a letter explaining why they are receiving this additional questionnaire and a FREEPOST envelope. They will be mailed one reminder after 2 weeks if the questionnaire is not returned.

After trial completion, a final audit of the GP electronic medical records will be run to identify all skin consultations for the 12 months during the trial as well as the previous 12 months.

8.7.3 Cancer Registry data

After trial completion, the regional Public Health England Knowledge and Intelligence Teams (KITs) will be given identifying data for all trial participants to identify any incident melanomas based upon the cancer data collected by the National Cancer Registration Service.

8.8 Measures

8.8.1 Patient Reported Outcome Measures (PROMs)

For all participants, these data will be collected at baseline, 6 months and 12 months post-trial consultation:

<u>a. Demographics and clinical variables</u>: age, gender, marital status, postcode, highest education level, occupation, past history of skin cancer (melanoma, SCC, BCC), skin and hair type (density of freckles on arms before age 20, natural hair colour at age 15, number of severe sunburns aged 2-18), number of raised moles on both arms, measured at baseline only.

<u>b. Sun protection habits scale</u>: developed by Glanz et al in the US for a multicomponent skin cancer prevention programme, this comprises 5 items measured using a 4 point Likert scale, and relating to use of sun protection, sun and sunbed habits, and episodes of sunburn in the previous year (27).

<u>c. Skin Self-Examination benefits and barriers scale:</u> validated by Manne and Lessin in the US among melanoma survivors, and developed from previous work on mammography and family members of patients with colorectal cancer (28). The benefits scale has seven items (α=.71) and the barriers scale has ten items (α=.74).

d. Melanoma Worry Scale (MWS): validated by Moye et al in the US (29), an adapted from the Breast Cancer Worry Scale (30), this measure comprises four items, scored 1 to 4, with possible scores ranging from 4 to 17, and higher scores indicating higher levels of worry.

e. Perceived Melanoma Risk: drawn from Manne and Lessin's measures (28), these two items have been widely used for melanoma and other cancer risk assessments to assess estimated percent risk of developing melanoma, and perceived risk compared with a person of the same age (relative risk).

f. Self-Efficacy for consulting without delay: A 10-item self-completed scale summed to score 10-100, was used in a primary care trial for lung symptoms, and showed good internal reliability (Cronbach α =0.85) (21, 31, 32). It has been adapted for this study, and reduced to 8 items.

g. Hospital anxiety and depression scale (HADS): This 14-item self-completed scale has been widely used to measure distress and has been extensively validated and shown to perform well in a wide range of populations (mean Cronbach α = 0.82; sensitivity and specificity 0.80) (33).

h. SF-12 Quality of life scale: a 12-item version of the SF-36 that is widely used and validated to measure functional health and well-being.

For all participants consulting their GP/practice nurse for skin changes/pigmented skin lesion during the 12 months following the trial consultation, their *Patient Interval (PI),* will be measured using the Symptom Study instrument modified for skin. This is a self-completed questionnaire to collect data on symptoms and their duration prior to consultation, and validated for lung, colorectal and pancreatic cancer symptoms (34).

8.8.2 Other Measures

a. Consultation Rates

Consultations for skin changes/pigmented skin lesions during the study period and 12 months before the trial will be identified by auditing the general practice electronic medical records. All actions taken during the consultation will be recorded including: diagnosis, referral, excision in the GP surgery, advice to monitor.

b. Trial feasibility and acceptability

As this is a phase II trial, we will obtain data on patient recruitment and attrition, and response rates to outcome measures to inform decisions about a future phase III trial.

c. Melanoma incidence in participating practices

This will be identified by data collected from primary care records.

8.8.3 Follow-up

We will obtain consent to follow-up the flagged participants in the National Cancer Registration Service after 5 years. We have recently undertaken this following our MoleMate trial, to look at melanoma and other skin cancer incidence post-completion of the trial.

8.9 Reimbursement

General practices will be reimbursed by CRN:Eastern to cover any administrative or time costs associated with supporting the study. Participants will be reimbursed for the purchase of the SSM App with an Apple Apps store/Google Play voucher.

8.10 Withdrawals and Protocol Deviations

Participants are able to withdraw from the study at any time. If a participant does decide to withdraw, we will emphasise that it will not in any way affect their car or their relationship with the general practice. This should also be mentioned when explaining the consent form.

Participants are also able to request removal of all their information/data collected during their participation in the study. If a participant would like to do this they are required to sign the withdrawal of participation form.

Deviations from the trial protocol will be recorded by the research staff. Deviations include, but are not restricted to, the following:

- Control participant given the SSM App and instruction booklet instead of just the standard information on skin cancer prevention;
- Questionnaire/s not completed;
- Questionnaire/s not completed in normal time period;
- Any other occasion whereby the above detailed instructions are not able to be adhered to or any reason.

All deviations should be recorded. The information will be used to inform a future phase III trial.

9. Data Management

9.1 Participant Data

All participants will be given a unique identifying code. The consent form will have both the participants name and code so they will be stored separately from the questionnaires in separate locked filing cabinets. If a participant wishes to withdraw their data from the project, the researchers will be able to link their ID code from their consent form. Forms and questionnaires will be stored in locked filing cabinets in a secure building, and data will be stored on a password protected computer at the University of Cambridge. Only the researchers on the study will have access to any data. Information will be aggregated in any publication to protect individuals from being identified.

9.2 Outcome Registry Intervention and Operation Network (ORION)

All information about the trial will be managed using a purpose built module hosted by ORION. This will be used to track participants through the study and collect data from the baseline and follow up questionnaires as they are completed. ORION is hosted and run by the University of Cambridge (Clinical Neurosciences) and it is overseen by a steering group comprised of University of Cambridge and NHS staff. It will host the MelaTools Q risk assessment questionnaire, the recruitment strategy validated in the Phase I study. Data are automatically validated and structured at the point of entry, and information stored within ORION is created, maintained, stored and managed accurately, with appropriate levels of security and accessibility, and safeguarded against inappropriate disclosure.

Information within ORION is handled in accordance with: The Data Protection Act 1998; The Freedom of Information Act 2000; ISO/IEC 27002:2005 Information Security Management; The NHS Information Security Code of Practice; The NHS Confidentiality Code of Practice; The NHS Records Management Code of Practice; The Caldicott Principles.

Compliance with information governance requirements set by the Department of Health is assessed and monitored by the NHS Connecting for Health Information Governance Toolkit. The algorithm for encryption of personal data (AES256) corresponds to the applicable NHS IG data encryption algorithm, and network connection encryption is in line with the group comprised of University of Cambridge and NHS staff and the Department of Health’s Secure Socket Layer (SSL) framework standard.

9.3 Study Management

Due to the non-medicinal and low-risk nature of the trial a data monitoring committee will not be needed. The trial steering committee (the CI, collaborators and researchers, statistician and PPIE representatives) will meet 6 monthly from the start of the study and will monitor study progress, approve a data analysis plan, and will ensure the study runs in accordance with the protocol and applicable standard operating procedures. The CI will take responsibility of data monitoring and ethics, and will be responsible for communicating important protocol modifications to relevant

parties. The trial is subject to the audit arrangements of the NIHR CRN. These are independent of the funder and the sponsor.

9.4 Sample Size and Power Calculation

Based on our recent MelaTools Q study with an identical screening step (response rate of 86%), we anticipate that we will need to approach approximately 2,000 people from 10 general practice waiting rooms to have about 1,600 complete the electronic questionnaire (23). About 25%, 400 people, will be identified as 'above-population' risk and be eligible to participate in the trial. Based on previous research we would expect approximately 50% of these to attend their trial consultation and undergo randomisation in order to reach our target of 200 participants.
We will use information on recruitment, retention, and data completeness, in addition to descriptive statistics for the primary and secondary outcomes measures, and preliminary estimates of effect sizes where appropriate, to inform the sample size calculation for a future phase III trial for this intervention where appropriate.

9.5 Data Analysis

All randomised patients will be considered eligible for inclusion in the analysis in accordance with the intention-to-treat analysis principle. As this is a phase II trial, fully describing and characterising the extent and nature of the missing data is an important part of the analysis. For the outcome analysis, appropriate methods for dealing with missing endpoint data will be detailed in the statistical analysis plan and be informed by a blinded review of the data. The baseline characteristics of the two arms will be described using summary statistics. Possible consent bias will be assessed by comparing demographic and clinical variables of participants against those who declined participation, and possible differential attrition will be assessed by comparing baseline characteristics of those who withdraw or die against those who remain in the study. These comparisons will be performed using a two sample t-test (or non-parametric equivalent) for continuous variables and chi-square test for categorical variables. The primary analysis will be a comparison between the two groups on the consultation rate for skin changes/pigmented skin lesion using a Poisson regression model. Patient Interval is expected to be right skewed. We will explore the nature and extent of the skewness and will consider it as a continuous variable in the analysis, but may explore other approaches, including transformation, categorisation, or methods for statistical inference based on bootstrap resampling, as appropriate. Comparisons between groups on continuous secondary endpoints will be undertaken using a linear model that includes the baseline value where applicable. Comparisons between groups on binary secondary endpoints will be performed using logistic regression. The analyses performed on the primary and secondary endpoints will be repeated adjusting for additional baseline covariates as part of a sensitivity analysis. Point estimates of the intervention effect will be presented with 95% confidence intervals and two-sided p-values. Unadjusted p-values from secondary analyses will be interpreted in proper context and be clearly labelled.

9.6 Outputs

The study findings will be disseminated at national and international meetings such as Society for Academic Primary Care (SAPC), and Cancer Primary Care Research International (CaPRI) meeting. The findings will be submitted to leading peer-reviewed scientific journals for publication, and will inform the design and implementation of a phase III trial on skin self-monitoring for people at higher risk for melanoma.

Participants will be able to view a summary of the results on the MelaTools website. On request, a paper copy of the summary sheet will be sent to the participant in the post.

10. References

1. Cancer Research UK website. Available from: http://www.cancerresearchuk.org/health-professional/cancer-statistics/mortality/common-cancers-compared#heading-Zero. Accessed September 2015.
2. Arnold M, Holterhues C, Hollestein LM, et al. Trends in incidence and predictions of cutaneous melanoma across Europe up to 2015. J Eur Acad Dermatol Venereol. 2014; 28:1170-8.
3. Diepgen T, Mahler V. The epidemiology of skin cancer. British Journal of Dermatology. 2002;146(s61):1-6.
4. Balch CM, Gershenwald JE, Soong S-j, Thompson JF, Atkins MB, Byrd DR, et al. Final version of 2009 AJCC melanoma staging and classification. Journal of Clinical Oncology. 2009;27(36):6199-206.
5. Cancer Research UK website. Available from: http://www.cancerresearchuk.org/cancer-info/cancerstats/types/skin/survival/skin-cancer-survival-statistics#one. Accessed September 2015.
6. Sant M, Allemani C, Santaquilani M, Knijn A, Marchesi F, Capocaccia R, et al. EUROCARE-4. Survival of cancer patients diagnosed in 1995–1999. Results and commentary. European Journal of Cancer. 2009;45(6):931-91.
7. Abdel-Rahman M, Stockton D, Rachet B, Hakulinen T, Coleman M. What if cancer survival in Britain were the same as in Europe: how many deaths are avoidable&quest. British journal of cancer. 2009;101:S115-S24.
8. Breitbart EW, Waldmann A, Nolte S, Capellaro M, Greinert R, Volkmer B, et al. Systematic skin cancer screening in Northern Germany. Journal of the American Academy of Dermatology. 2012;66(2):201-11.
9. Baughan P, O'Neill B, Fletcher E. Auditing the diagnosis of cancer in primary care: the experience in Scotland. British journal of cancer. 2009;101:S87-S91.
10. Richards MA. The size of the prize for earlier diagnosis of cancer in England. British Journal of Cancer. 2009;101 Suppl 2:S125-9. Epub 2009/12/04.
11. Rubin G, Vedsted P, Emery J. Improving cancer outcomes: better access to diagnostics in primary care could be critical. Br J Gen Pract. 2011;61(586):317-8. Epub 2011/05/31.
12. Walter F, Webster, A. Scott, S. and Emery, J. . The Andersen Model of Total Patient Delay: a systematic review of its application in cancer diagnosis. . Journal of Health Service Research Policy 2011;10:1-13.
13. Walter FM, Humphrys E, Tso S, et al. Patient understanding of moles and skin cancer, and factors influencing presentation in primary care: a qualitative study. BMC Fam Pract 2010;11:62.
14. Walter FM, Birt L, Cavers D. et al. 'This isn't what mine looked like': a qualitative study of symptom appraisal and help seeking in people recently diagnosed with melanoma. BMJ Open 2014; 4(7) e005566. Epub 2014/07/24
15. Senior K. Smart phones: new clinical tools in oncology? The lancet oncology. 2011;12(5):429-30.
16. Boudreaux ED, Waring ME, Hayes RB, Sadasivam RS, Mullen S, Pagoto S. Evaluating and selecting mobile health apps: strategies for healthcare providers and healthcare organizations. Translational behavioral medicine. 2014;4(4):363-71.
17. Korner A, Coroiu A, Martins C. et al. Predictors of skin self-examination before and after a melanoma diagnosis: the role of medical advice and patient's level of education. Int Arch Med 2013; 6(1):8.
18. Hamidi R, Peng D, Cockburn M. Efficacy of skin self-examination for the early detection of melanoma. Int J Dermatol. 2010; 49(2): 126-34.
19. Carli P, De Giorgi V,Palli D et al. Dermatologist detection and skin self-examination are associated with thinner melanomas: results from a survey of the Italian Multidisciplinary Group on Melanoma. Arch Dermatol. 2003; 139(5):607-12.
20. Korner A, Drapeau M, Thombs BD, et al. Barriers and facilitators of adherence to medical advice on skin self-examination during melanoma follow-up care. BMC Dermatol 2013; 13:3.

21. Murchie P, Allan JL, Brant W, Dennis M, Hall S, Masthoff J, et al. Total skin self-examination at home for people treated for cutaneous melanoma: development and pilot of a digital intervention. BMJ open. 2015;5(8):e007993.

22. Williams LH, Shors AR, Barlow WE, Solomon C, White E. Identifying Persons at Highest Risk of Melanoma Using Self-Assessed Risk Factors. J Clin Exp Dermatol Res 2011;2:pii:1000129, doi:10.4172/2155-9554.1000129.

23. Usher-Smith JA, Kassianos AP, Emery JD, Abel GA, Teoh Z, Hall S, Neal RD, Murchie P, Walter FM. Identifying people at higher risk of melanoma across the UK: a primary care-based electronic survey. In submission to JAMA Dermatology

24. Campbell NC, Murray E, Darbyshire J, Emery J, Farmer A, Griffiths F, et al. Designing and evaluating complex interventions to improve health care. Bmj. 2007;334(7591):455-9. Epub 2007/03/03.

25. Craig P, Dieppe P, Macintyre S, Michie S, Nazareth I, Petticrew M. Developing and evaluating complex interventions: the new Medical Research Council guidance. BMJ 2008;337:a1655. Epub 2008/10/01.

26. Hardeman W, Lamming L, Kellar I et al. Implementation of a nurse-led behaviour change intervention to support medication taking in type 2 diabetes: beyond hypothesised active ingredients (SAMS Consultation Study) Implement Sci 2014; 9:70.

27. Glanz K, Gelle AC, Shigaki D, Maddock JE, Isnec MR. A randomized trial of skin cancer prevention in aquatics settings: the Pool Cool program. Health Psychol. 2002; 21(6):579-587.

28. Manne S, Lesin S. Prevalence and correlates of sun protection and skin self-examination practices among cutaneous malignant melanoma survivors. J Behav Med. 2006; 29(5):419-34.

29. Moye MS, King SM, Rice ZP, DeLong LK, Seidler AM, Veledar E, Curiel-Lewandrowski C, Chen SC. JAMA Dermatol. 2015; 151(2):137-43.

30. Lerman C, Trock B, Rimer BK, Jepson C, Brody D, Boyce A. Psychological side effects of breast cancer screening. Health Psychol. 1991;10(4):259-267/

31. Smith S, Fielding S, Murchie P, Johnston M, Wyke S, Powell R, et al. Reducing the time before consulting with symptoms of lung cancer: a randomised controlled trial in primary care. Br J Gen Pract. 2013;63(606):47-54. Epub 2013/01/23.

32. Smith SM, Murchie P, Devereux G, Johnston M, Lee AJ, Macleod U, et al. Developing a complex intervention to reduce time to presentation with symptoms of lung cancer. Br J Gen Pract. 2012;62(602):e605-15. Epub 2012/09/06.

33. Zigmond AS, Snaith RP. The hospital anxiety and depression scale. ACTA Psychiatr Scand 1983; 67:361-70.

34. Ware, J, Kosinski M, Keller SD. A 12-Item Short-Form Health Survey: construction of scales and preliminary tests of reliability and validity. Med Care 1996; 34(3):220-233.

35. Walter FM, Rubin G, Bankhead C, Morris HC, Hall N, Mills K, Dobson C, Rintoul RC, Hamilton W, Emery J. Symptoms and other factors associated with time to diagnosis and stage of lung cancer: a prospective cohort study. BJC 2015; 112 Suppl 1:S6-13.

MelaTools-SSM Statistical Analysis Plan

Contents

1. Administrative information 2
   - Trial title 2
   - Trial registration 2
   - SAP revision history 2
   - Protocol version 2
   - Roles and responsibility 3
2. Introduction 4
   - Aims 4
   - Hypotheses 4
3. Methods 5
   - Design 5
   - Sample size 5
   - Framework 5
   - Interim analyses and stopping guidance 5
   - Timing of final analysis 5
   - Outcomes 5
4. Statistical Principles 7
   - Confidence intervals and p-values 7
   - Adherence and protocol deviations 7
   - Analysis population 7
5. Trial population 8
   - Screening data 8
   - Eligibility 8
   - Recruitment 8
   - Baseline characteristics 8
6. Analysis 10
   - Outcome definitions 10
   - Analysis methods 12
   - Harms 17
   - Statistical software 17
   - Additional analyses 17

References 18

1. ADMINISTRATIVE INFORMATION

Trial title

MelaTools Skin Self-Monitoring Trial: a phase II randomised controlled trial of an intervention for primary care patients at higher risk of melanoma

Trial registration

ISCTRN16061621

SAP revision history

Version 1.2

Last edited 1st February 2019 by Matt Barclay.

Protocol version	Updated SAP version number	Section number changed	Description of and reason for change	Date changed
Published (Mills *et al*, 2017)	1	NA	First version	25th January 2017
Published (Mills *et al*, 2017)	1.1	Section 6 and Appendix B	Adding scoring rules and table	29th January 2017
Published (Mills *et al*, 2017)	1.2	Section 6	Noting that the secondary outcome "Have you ever used a sunbed?" was not analysed	1 February 2019

Protocol version

This document has been written based on information contained in the study protocol (Mills *et al*, 2017).

Roles and responsibility

Matthew E Barclay is the trial statistician and drafted the statistical analysis plan.

Catherine L Saunders is the responsible senior statistician and critically reviewed and revised the SAP, and takes final responsibility for the content.

Fiona M Walter is the chief investigator.

MEB, CLS and FMW agreed the final version

Signature	Role	Date
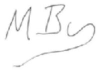	Trial statistician	25th Jan 2017
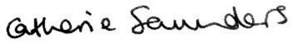	Senior statistician	28th Dec 2017
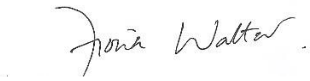	Chief investigator	24th Jan 2018

2. INTRODUCTION

This phase II feasibility randomised controlled trial (RCT) tests a novel intervention of the use of a Skin Self-Monitoring (SSM) smartphone application (‘App’), and self-monitoring monthly reminders, to promote timely consulting for skin changes/pigmented skin lesions by people at above-population risk of melanoma.

Aims

- To assess the effect of using a SSM App compared with standard information about detecting skin cancer on the patient interval (time from first noticing skin change to consultation) among patients at above-population risk of melanoma;
- To obtain preliminary estimates of effect across a range of outcome measures to inform selection of the primary outcome for a definitive phase III trial.

Hypotheses

- The MelaTools SSM Trial intervention will increase the number of consultations for pigmented skin lesions in people at above-population risk of melanoma.
- The MelaTools SSM Trial intervention will be acceptable and will reduce the patient interval (including symptom appraisal and help-seeking intervals) in people at above-population risk of melanoma.
- The MelaTools SSM Trial intervention will not cause significant distress or worry in people at above population risk of melanoma.

3. METHODS

Design

The phase II trial is a multisite individually randomised controlled trial set in 12 general practices across Eastern England. Those who meet the eligibility criteria and consent to participate are randomised 1:1 into either the control or intervention group. Randomisation is being performed using an online system provided by the Clinical Trials Unit based at Kings College London.

Sample size

The sample size for this Phase II trial is 200. 2000 people will be approached in order to find 400 high risk patients, of whom around 200 are expected to consent, as detailed in the trial protocol (Mills *et al*, 2017).

Framework

This trial is a superiority trial. Comparisons will be presented on this basis. This is a phase II feasibility trial and is not powered as a superiority trial for the primary outcome. Estimates will inform the future phase III trial.

Interim analyses and stopping guidance

No interim analyses are planned.

Timing of final analysis

All outcomes will be analysed collectively at the end of the trial.

Outcomes

Primary outcomes

Consultation rates

Consultation rates for any skin changes/pigmented skin lesions presented to their GP/practice nurse during the 12 months following the trial consultation compared with the 12 months prior to the trial.

The patient interval

The patient interval for all skin changes/pigmented skin lesions presented to their GP/practice nurse during the 12 months following the trial consultation.

Patient interval measures will be collected in questionnaires sent to patients who attend their GP for skin changes/pigmented skin lesions.

Secondary outcomes

Patient-reported outcomes

Sun protection habits, skin self-examination, melanoma worry and perceived melanoma risk, self-efficacy for consulting without delay; anxiety, depression and quality of life.

These will be collected in questionnaires at baseline, 6 months following randomisation and 12 months following randomisation. Baseline questionnaires are collected after consent at the practice using an i-pad. Participants enter their data electronically on the i-pad which linked to a secure database where the data will be stored. For the 6- and 12-month follow-up patients will be sent an email with a secure link connecting them to the database. This will allow them to complete the questionnaires safely and electronically. If a participant indicates they would prefer a hardcopy they will be sent one, including a cover letter and a FREEpost return envelope. For the 12-month follow-up, if a participant has not responded, they will be called to see if they would like to complete the questionnaire, either by electronic link, hardcopy, or over the phone.

Trial feasibility and acceptability

Data on patient recruitment, attrition, and response rates to outcome measures to inform decisions about a future phase III trial.

Melanoma incidence

Melanoma incidence across participating practices, to contextualise trial findings.

4. STATISTICAL PRINCIPLES

Confidence intervals and p-values

All applicable statistical tests will be 2-sided and will be performed using a 5% significance level. No corrections for multiple testing will be made. Where appropriate, 95% confidence intervals will be reported.

Adherence and protocol deviations

Adherence

Adherence to the intervention is not monitored. Those who are adherent to the intervention are those who are using the skin self-monitoring (SSM) app regularly (once a month or more frequently). People in the intervention group receive monthly reminders to use the SSM app. Follow-up questionnaires do not include questions on use of the SSM app.

Protocol deviations

Protocol deviations will be classified prior to unblinding of treatment. The number (and percentage) of patients with major and minor protocol deviations will be summarised by treatment group with details of type of deviation provided. The patients that are included in the ITT analysis data set will be used as the denominator to calculate the percentages. No formal statistical testing will be undertaken.

Use of other SSM apps

We will describe the apps and the intensity of use, overall and by trial arm

Differential follow-up

Data quality checks will focus on any practices without any reported consultations in their patients, and on any patients with a large number of reported consultations in the year after randomization. It may not be possible to check all practices and patients.

We will describe the number of practice and patients checked, and any resultant changes to the analysis dataset. All changes will happen before the treatment allocation is unblended.

Analysis population

All analysis will be intention-to-treat, including all randomised patients according to the treatment they were randomised to receive.

5. TRIAL POPULATION

Screening data

We will report the number of patients assessed for eligibility before recruitment.

Eligibility

Inclusion criteria

People aged between 18 and 75 years who own a smartphone (Apple or Android), and, on completion of the Melatools-Q risk assessment tool (Williams *et al*, 2011), are found to be at increased risk of melanoma. Participants are able to read and write English and to give informed consent.

Exclusion criteria

Previous diagnosis of melanoma, severe psychiatric or cognitive disorders, or a physical disorder severe enough to inhibit the use of a smartphone.

Recruitment

We will report a CONSORT patient flow diagram, including the number of patients

- assessed for eligibility at screening
- eligible at screening
- ineligible at screening *
- eligible and randomised
- eligible but not randomised *
- received the randomised allocation
- did not receive the randomised allocation *
- lost to follow-up *
- discontinued the intervention *
- randomised and included in the primary analysis
- randomised and excluded from the primary analysis *

***** reasons will be provided

Specifically, for this analysis, we will report in full

- response rates to each of the PRO surveys (baseline, 6 and 12 months)
- response rates to the patient interval surveys

We will also report

- item non-response to each survey item among responders
- Timings for any withdrawals

Baseline characteristics

We will provide summary statistics on baseline characteristics overall and by arm.

We will describe the median (IQR) consultation rate in the year before randomisation, as well as the median (IQR) age at randomisation and the proportion female. We will further describe the mean (standard deviation) of each of the 9 scale secondary outcome measure, and the baseline distribution of the additional secondary measures which do not form part of a scale.

6. ANALYSIS

Outcome definitions

Primary outcomes

Consultation rate
The number of consultations for skin changes/pigmented skin lesions per person in the 12 months following randomisation.

This will be collected monthly by a search of general practice records. Practices with zero recorded consultations at the end of the trial will be higher priority for visits to check for failure to report data. The aspiration is to visit all 12 practices to search for consultations that have not been reported, but this may not be practical given time constraints.

This will initially be reported as “patient [patientID] consulted for pigmented skin lesions on [date]” and will be converted to a rate (consultations per patient per year) for analysis. We will have baseline data on consultations in the 12 months prior to randomisation.

Patient interval
The number of days between detection of skin changes/pigmented skin lesions and presentation to GP / practice nurse for consultations in the 12 months following randomisation.

This will be collected via a questionnaire (‘skin questionnaire’). Patients’ visit to their GP or practice nurse will trigger the sending of these questionnaires. Non-responders will be sent a reminder questionnaire and may receive a telephone call from trial staff to ask them to complete the information.

Patient questionnaires ask about several specific symptoms and the first time a patient experienced it (if they did), and when they first approached their GP or practice nurse about this symptom, and has a free-text box for other symptoms. We will define the patient interval as the time from the first reported symptom until the first reported time they approached their GP or practice nurse.

For example, if a patients’ first symptom was an irregular shaped mole but the first time they told their GP or nurse was about a different symptom (for example, inflammation) then we would define the patient interval as “date told GP about inflammation” – “date noticed irregular shaped mole”.

Free text responses will be checked by the trial statistician and reviewed (blind to treatment allocation) by the principal investigator, to ensure that reported symptoms are actually possible symptoms of melanoma.

Secondary outcomes
We have 9 scale items (see Appendix B. Scoring rules for the survey instruments.).

1. Social Factors Inventory 12 (Ware *et al*, 1998), Physical Component Summary (PCS)
    - Higher score = better physical health, range 0 to 100
2. Social Factors Inventory 12, Mental Component Summary (MCS)
    - Higher score = better mental health, range 0 to 100

3. Sun Protection Habits (Glanz *et al*, 2002)
    - Higher score = better sun protection habits, range 1-4 (mean of Qs)
4. Melanoma Worry Scale (Moye *et al*, 2015)
    - Higher score = more worry, range 4-17 (sum of Qs)
5. Self-efficacy for consulting without delay (Smith *et al*, 2012; Smith *et al*, 2013)
    - Higher score = more confident about consulting quickly, range 8-80 (sum of Qs)
6. HADS-D (Depression) (Zigmond and Snaith, 1983)
    - Higher score = more depressed, range 0-21 (sum of Qs)
7. HADS-A (Anxiety)
    - Higher score = more anxious, range 0-21 (sum of Qs)
8. Skin Self-Examination Benefits (Manne and Lessin, 2006)
    - Higher score = more benefits, range 7-35 (sum of Qs)
9. Skin Self-Examination Barriers
    - Higher score = more barriers, range 10-50 (sum of Qs)

There are several additional secondary outcomes which will be analysed on their own, rather than as scales.

- Did you practice sun protection last year?
    - "Rarely or never" , … , "Always"
- How likely are you to practice sun protection in the coming year?
    - "Not at all likely", … , "Extremely likely"
- Have you ever used sunbeds?
    - "Yes" / "No"
    - EDIT – 1 February 2019 – this outcome was excluded from analysis.
- How many times have you been sunburnt in the last year?
    - 0, 1-2, "3-5", "More than 5"
- Compared with other people, do you think your chances of getting melanoma are lower or higher?
    - "Much lower" , … , "Much higher"
- What do you think your lifetime risk of melanoma is?
    - 0% to 100%
- Have you downloaded any apps [for detecting melanoma]?
    - "No" / "Yes"
- If yes, name?
    - [freetext]
- How often have you used this app?
    - "Never" , "Once", "Once every 6 months", "Once every 3 months", "Every month"

We will describe the distribution of responses to these questions overall and by arm, at baseline, 6 months follow-up and 12 months follow-up.

These will be collected from patients using questionnaires sent out at baseline (pre-randomisation), 6 months and 12 months follow-up.

Those who do not respond to the follow-up questionnaire initially will receive a reminder email, and a phone call reminder.

Analysis methods

Analysis of possible consent bias

Possible consent bias will be assessed by comparing demographics (age, sex) of participants against those who declined participation. These comparisons will be performed using a two sample t-test for continuous variables and chi-square test for categorical variables.

Analysis of possible differential attrition

Possible differential attrition will be assessed by comparing baseline characteristics of those who withdraw or die against those who remain in the trial. These comparisons will be performed using a two sample t-test for continuous variables and chi-square test for categorical variables.

Analysis of non-response

We will compare baseline characteristics of those who do not respond to follow-up questionnaires and to patient interval questionnaires with those who do respond. These comparisons will be performed using a two sample t-test for continuous variables and chi-square test for categorical variables.

Analysis of consultation rate

Data quality checks

- Plot a boxplot of all the consultation rates
- Plot a histogram of all the consultation rates
  - *Looking for extreme values, a priori defined as people with 4 or more consultations for suspected skin cancer in a 12 month period. These will be checked for accuracy with the relevant general practice, and either corrected or confirmed accurate and kept in.*
- Plot a boxplot of all the consultation rates, by general practice
  - *Looking for general practices with oddly high or low consultation rates (although noting that we do expect some general practices to have 0 consultations). The concern will be if there are some practices with high rates across the board, and others with low rates across the board – that may require following up on.*

Analysis planning

The protocol specifies Poisson regression for this outcome. This is the standard approach for count data. It is possible that there is over-dispersion in consultation rates and that other approaches such as negative binomial regression would be better. If this is the case, then it will be important to use a more appropriate analysis method in the phase III trial. We will report Pearson over-dispersion statistics to inform analysis choice in the phase III trial. For this analysis, we will use Poisson regression.

Missing data

There should not be missing data in this outcome as we assume zero consultation rates are true zeros.

Statistical analysis

First, we will describe the pre- and post- intervention consultation rates overall and by group, summarising using median (IQR) and mean (SD). We will not perform a statistical test, but this will be useful information for sample size calculations for the phase III trial.

The main analysis will be an unadjusted Poisson regression.

$$Consultation\ rate, post = \exp(\beta_0 + \beta_1 \times (Intervention))$$

In Stata code

```
glm consult_post i.treat, family(poisson)
```

A sensitivity analysis will adjust for demographic information and include a random effect for general practice.

$$Consultation\ rate, post = \exp(\beta_0 + \beta_1 \times (Intervention) + \beta_2 \times (Female) + \beta_3 \times (Age\ in\ years) + b_1)$$

Where $b_1 \sim N(0, \sigma_1^2)$ is a random effect to capture GP effects.

In Stata code

```
meglm consult_post i.treat i.female c.age, family(poisson) || practice:
```

We will explore the impact of allowing for non-linear associations with age, by introducing a changepoint at 65 years.

Analysis of patient interval among patients who have had a consultation

Data quality checks

- Plot a boxplot of all the patient intervals
- Plot a histogram of all the patient intervals
    - *We know from Lyratzopoulos et al (2015) that the quartiles of the patient interval for a different sample of patients with melanoma were Min: 0, Q1: 0, Q2: 21, Q3: 69, pct90: 234 days. We will have concerns about either data quality or generalisability if more than 25% of patients have a patient interval of 80+ days.*

Analysis planning

The protocol specifies standard linear regression, and includes flexibility on the particular analysis in case data are highly skewed.

We will use linear regression to analyse these data. We will also tabulate consultations by trial arm and whether the patient interval was more (or less) than 21 days.

Missing data

We expect missing data in this outcome, because it is based on a survey of patients and not all patients will respond.

In the first instance, we will compare rates of missing data between the intervention and control groups. If these rates are similar (within ten percentage points) we will not try to adjust for missing data. We will not report statistical significance if the arms have different rates of missing data. So, 42% and 50% missing we will report, but 39% and 50% missing we will not.

We will compare baseline demographics of responders and non-responders as described in 'Analysis of non-response' (Page 12).

Statistical analysis

The rate of missing data will be an important outcome. High rates of missing data suggest this will not be a useful measure in a phase III trial, or that the trial will need to include specific guidelines for the missing data and be powered to appropriately.

We will describe the median (IQR) and mean (SD) patient intervals overall and by intervention group, without performing a statistical test. We will also describe the number and proportion of consultations with patient interval more (or less) than 21 days by trial arm.

The main analysis will be an unadjusted multilevel linear regression:

$$PatientInterval_{ij} = \beta_0 + \beta_1 \times (Intervention_i) + b_i + \epsilon_{ij}$$

Where $b_i \sim N(0, \sigma_2^2)$ is a patient-level random effect and $\epsilon_{ij} \sim N(0, \sigma^2)$ is the error term for the *j*th consultation the *i*th patient has. If all patients have at most one set of patient interval information, we will use standard linear regression.

Sensitivity analyses will adjust for sex and age.

$$PatientInterval_{ij} = \beta_0 + \beta_1 \times (Intervention_i) + \beta_2 \times (Female) + \beta_3 \times (Age\ in\ years) + b_i + \epsilon_{ij}$$

We will explore the impact of allowing for non-linear associations with age, by introducing a changepoint at 65 years.

Analysis of secondary outcomes – the nine scale items

These are described in Section 6. Analysis, on page 10. Information on these secondary outcomes was collected at baseline, 6 months and 12 months follow-up.

Data quality

The SF-12 scoring manual recommends several data quality checks to make sure the scoring is correct.

- Check correlations between physical functioning, role physical and pain questions and the SF12 PCS (should be high) and SF12 MCS (should be low).
- Check correlations between social functioning, role emotional and mental health questions and the SF12 PCS (should be low) and SF12 MCS (should be high)

- Check correlation between PCS and MCS (should be low)

We will additionally plot boxplots and histograms of each set of scale, looking for "out of range" errors (to check for coding mistakes) and to check the distributions appear as expected.

Missing data

We have two types of missing data here. The first is where a patient did not return a questionnaire. The second is where a patient did not fully complete a questionnaire. Analysis of those who did not return questionnaires is described on page 12. We will further describe item-level non-response for each individual question by trial arm.

With the exception of the Sun Protection Habits scale (where a valid score can apparently be produced when one or two of the five questions are blank (Glanz *et al*, 2002)), none of these scales can handle missing data. There is a standard imputation process for SF12 which we have not implemented for this analysis.

We will compare the rates of scale-level missing data by trial arm. As for patient interval, we will not report p-values where rates of missing data differ by more than 10%-points between trial arms.

Statistical analysis

First, we will report the proportion of missing data on each scale (both (a) proportion for whom scale could not be produced and (b) patient-level average proportion of Qs missing).

We will describe the mean (SD) of each scale overall and by intervention group at 6 and 12 months follow-up, without performing a statistical test.

We will analyse these secondary outcomes using separate multilevel linear regression models for each scale.

$$Score_{i,t} = \beta_0 + \beta_1 \times (Score_{i,0}) + \beta_2 \times (Intervention_i) + b_{1,i} + \epsilon_{i,t}$$

for patient *i* at time *t* (as we will have responses at 0, 6 and 12 months follow-up), with $b_{1,i} \sim N(0, \sigma_1^2)$ and $\epsilon_{i,t} \sim N(0, \sigma^2)$.

We will then investigate the adjusted difference as a sensitivity analysis,

$$Score_{i,t} = \beta_0 + \beta_1 \times \left(Score_{i,0}\right) + \beta_2 \times (Intervention_i) + \beta_3 \times (Female) + \beta_4 \times (Age\ in\ years) + b_{1,i} + \epsilon_{i,t}$$

We will additionally explore whether the impact changed over time by introducing a dummy variable for whether a questionnaire was from 6 or 12 months follow-up, with an interaction with the intervention.

Analysis of secondary outcomes – others

There are several other secondary outcomes which do not form part of scales.

Statistical analysis and missing data

Analysis for these items will be entirely descriptive. We will quantify the amount of missing data, and then describe the distribution of these responses at 0,6,12 months follow-up by trial arm (without any statistical testing).

If we do see apparent differences then this may suggest that one of these is a good outcome variable for use in the Phase III.

We will not present p-values for differences on these outcomes.

Analysis of melanoma incidence

This is just for context, and of course is a useful data quality check.

We will request from PHE ODR the full count of incident cases across these practices in 2013 to 2015 and nationally, without any demographic information. We will then calculate the rate as the total number of cases divided by the total practice list size / population of England.

This avoids any identifiability concerns around patients registered at these practices who have not consented to take part in the trial.

Harms

No specific adverse event information has been collected for this trial, beyond outcome measures already described.

Statistical software

All analysis will be carried out in Stata v15.

Additional analyses

We have described several exploratory analyses looking for evidence of differential impact to help in design of the analysis for the phase III trial.

REFERENCES

Glanz K, Geller AC, Shigaki D, Maddock JE and Isnec MR (2002) A randomized trial of skin cancer prevention in aquatics settings: the Pool Cool program. *Health Psychol* **21**: 579-87,

Lyratzopoulos G, Saunders CL, Abel GA, Mcphail S, Neal RD, Wardle J and Rubin GP (2015) The relative length of the patient and the primary care interval in patients with 28 common and rarer cancers. *Br J Cancer* **112**: S35-S40, doi:10.1038/bjc.2015.40

Manne S and Lessin S (2006) Prevalence and correlates of sun protection and skin self-examination practices among cutaneous malignant melanoma survivors. *J Behav Med* **29**: 419-34, doi:10.1007/s10865-006-9064-5

Mills K, Emery J, Lantaff R, Radford M, Pannebakker M, Hall P, Burrows N, Williams K, Saunders CL, Murchie P and Walter FM (2017) Protocol for the melatools skin self-monitoring trial: a phase II randomised controlled trial of an intervention for primary care patients at higher risk of melanoma. *BMJ Open* **7**, doi:10.1136/bmjopen-2017-017934

Moye MS, King SM, Rice ZP, Delong LK, Seidler AM, Veledar E, Curiel-Lewandrowski C and Chen SC (2015) Effects of total-body digital photography on cancer worry in patients with atypical mole syndrome. *JAMA Dermatol* **151**: 137-43, doi:10.1001/jamadermatol.2014.2229

Smith S, Fielding S, Murchie P, Johnston M, Wyke S, Powell R, Devereux G, Nicolson M, Macleod U, Wilson P, Ritchie L, Lee AJ and Campbell NC (2013) Reducing the time before consulting with symptoms of lung cancer: a randomised controlled trial in primary care. *Br J Gen Pract* **63**: e47-54, doi:10.3399/bjgp13X660779

Smith SM, Murchie P, Devereux G, Johnston M, Lee AJ, Macleod U, Nicolson MC, Powell R, Ritchie LD, Wyke S and Campbell NC (2012) Developing a complex intervention to reduce time to presentation with symptoms of lung cancer. *Br J Gen Pract* **62**: e605-15, doi:10.3399/bjgp12X654579

Ware J, A. Kosinski M and D. Keller S (1998) *SF-12: How to Score the SF-12 Physical and Mental Health Summary Scales.*

Williams LH, Shors AR, Barlow WE, Solomon C and White E (2011) Identifying Persons at Highest Risk of Melanoma Using Self-Assessed Risk Factors. *J Clin Exp Dermatol Res* **2**, doi:10.4172/2155-9554.1000129

Zigmond AS and Snaith RP (1983) The hospital anxiety and depression scale. *Acta Psychiatr Scand* **67**: 361-70,

APPENDIX A. PRELIMINARY TABLES AND FIGURES

Methods and baseline characteristics

Figure 1. Consort patient flow diagram

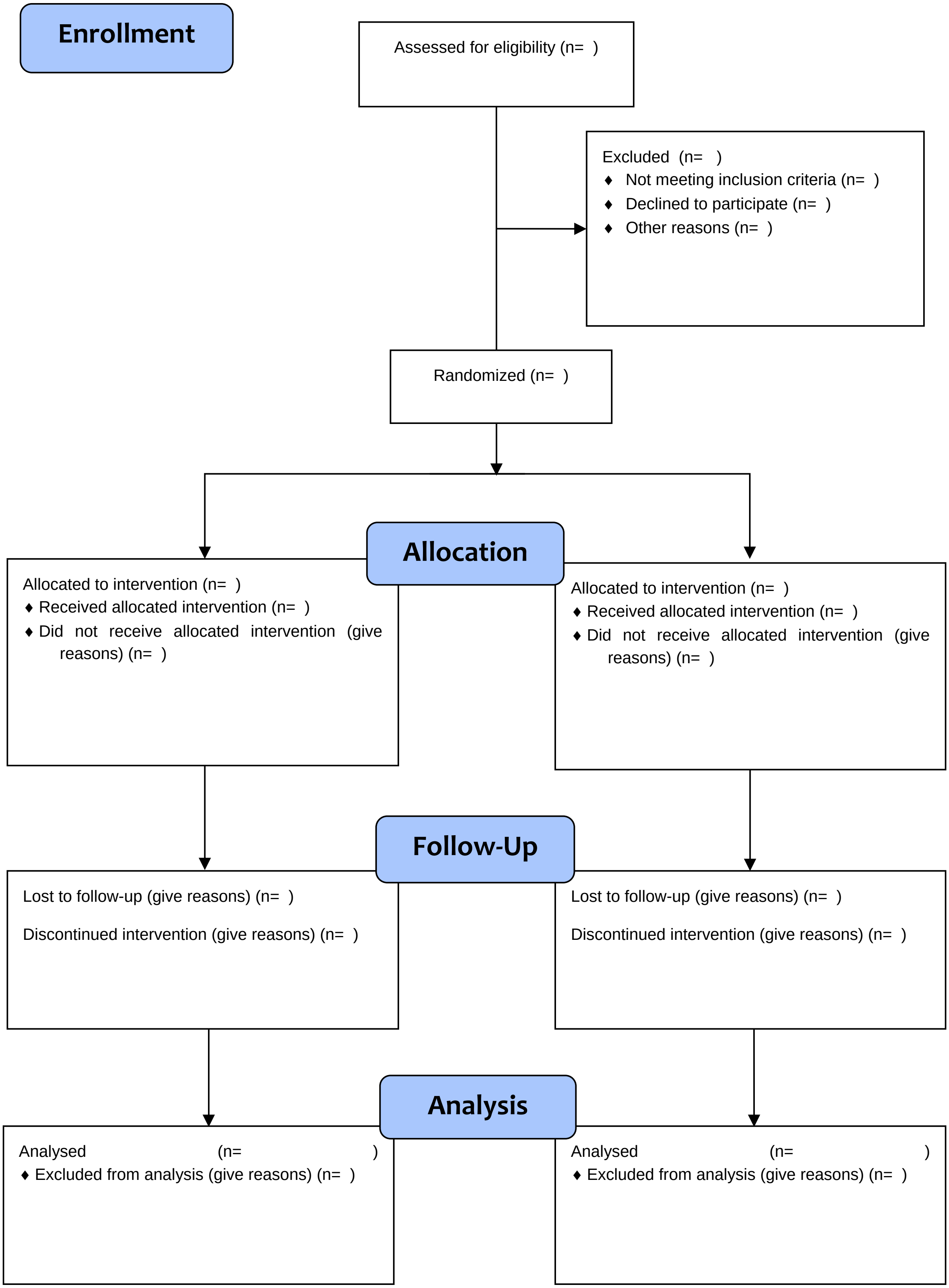
Enrollment
Assessed for eligibility (n=)
Excluded (n=)
♦ Not meeting inclusion criteria (n=)
♦ Declined to participate (n=)
♦ Other reasons (n=)
Randomized (n=)
Allocation
Allocated to intervention (n=)
♦ Received allocated intervention (n=)
♦ Did not receive allocated intervention (give reasons) (n=)
Allocated to intervention (n=)
♦ Received allocated intervention (n=)
♦ Did not receive allocated intervention (give reasons) (n=)
Follow-Up
Lost to follow-up (give reasons) (n=)
Discontinued intervention (give reasons) (n=)
Lost to follow-up (give reasons) (n=)
Discontinued intervention (give reasons) (n=)
Analysis
Analysed (n=)
♦ Excluded from analysis (give reasons) (n=)
Analysed (n=)
♦ Excluded from analysis (give reasons) (n=)

Table 1. Baseline characteristics of patients in the trial, overall and by trial arm

		Overall	Arm A	Arm B
Pre-trial consultation rate	*median (IQR)*			
Age at randomisation	*median (IQR)*			
Female	*N (proportion)*			
Social factors inventory 12, Physical component summary	*mean (SD, N)*			
Social factors inventory 12, Mental component summary	*mean (SD, N)*			
Sun protection habits score	*mean (SD, N)*			
Melanoma worry scale	*mean (SD, N)*			
Self-efficacy for consulting without delay	*mean (SD, N)*			
HADS-D Depression score	*mean (SD, N)*			
HADS-A Anxiety score	*mean (SD, N)*			
Skin self examination benefits score	*mean (SD, N)*			
Skin self-examination barriers score	*mean (SD, N)*			
Did you practice sun protection last year?	*Responses*<br><br>Rarely or never<br>Sometimes<br>Often<br>always			
How likely are you to practice sun protection in the coming year?	*Responses*<br><br>Not at all likely<br>Unlikely<br>Neither likely nor unlikely<br>Likely<br>Extremely likely			
Ever used sunbeds	*N (proportion)*			
How many times have you been sunburnt in the last year?	*Responses*<br><br>0<br>1-2<br>3-5<br>More than 5			
Compared with other people, do you think your chances of getting melanoma are lower or higher?	*Responses*<br><br>Much lower<br>Lower<br>Neither higher nor lower<br>Higher<br>Much higher			

		Overall	Arm A	Arm B
What do you think your lifetime risk of melanoma is? (%)	*Mean (SD)*			
Have you downloaded any apps for detecting melanoma?	*Responses*<br><br>*Proportion Yes (N)*			
How often have you used this app?	*Responses*<br><br>Never<br>Once every 6 months<br>Once every 3 months<br>Every month			

Intervention results

Table 2. Missing data on patient interval and secondary outcomes overall and by arm

**Outcome**	**Follow-up questionnaire**	**Overall**		**Arm A**		**Arm B**		**No statistical testing**
		**Possible**	**Missing (proportion)**	**Possible**	**Missing (proportion)**	**Possible**	**Missing (proportion)**	
**Patient interval**								
**Social factors inventory 12, Physical component summary**	**Both**							
	**6 month**							
	**12 month**							
**Social factors inventory 12, Mental component summary**	**Both**							
	**6 month**							
	**12 month**							
**Sun protection habits score**	**Both**							
	**6 month**							
	**12 month**							
**Melanoma worry scale**	**Both**							
	**6 month**							
	**12 month**							
**Self-efficacy for consulting without delay**	**Both**							
	**6 month**							

	**12 month**				
**HADS-D Depression score**	**Both**<br><br><br>**6 month**<br>**12 month**				
**HADS-A Anxiety score**	**Both**<br><br><br>**6 month**<br>**12 month**				
**Skin self examination benefits score**	**Both**<br><br><br><br>**6 month**<br>**12 month**				
**Skin self-examination barriers score**	**Both**<br><br><br><br>**6 month**<br>**12 month**				

Table 3. Results of regression analysis of consultation rate and of patient interval

**Consultation rate (Poisson regression)**	*Pearson overdispersion:*	**IRR**	**(95% CI)**	**p-value**
	Intervention			
	Intercept			
**Patient interval (multilevel linear regression)**		**Mean difference**	**(95% CI)**	**p-value**
	Intervention			
	Intercept			
	Patient-level random effect SD			

Table 4. Results of regression analysis of secondary outcome scales

**Outcome**	**Coefficient**	**Mean difference**	**(95% CI)**	**p-value**
**Social factors inventory 12, Physical component summary**				
	Intervention			
	Baseline score			
	Intercept			
	Patient-level random effect SD			
**Social factors inventory 12, Mental component summary**				
	Intervention			
	Baseline score			
	Intercept			
	Patient-level random effect SD			
**Sun protection habits score**				
	Intervention			
	Baseline score			
	Intercept			
	Patient-level random effect SD			
**Melanoma worry scale**				
	Intervention			
	Baseline score			
	Intercept			
	Patient-level random effect SD			
**Self-efficacy for consulting without delay**				
	Intervention			
	Baseline score			

Outcome	Coefficient	Mean difference	(95% CI)	p-value
	Intercept			
	Patient-level random effect SD			
**HADS-D Depression score**				
	Intervention			
	Baseline score			
	Intercept			
	Patient-level random effect SD			
**HADS-A Anxiety score**				
	Intervention			
	Baseline score			
	Intercept			
	Patient-level random effect SD			
**Skin self examination benefits score**				
	Intervention			
	Baseline score			
	Intercept			
	Patient-level random effect SD			
**Skin self-examination barriers score**				
	Intervention			
	Baseline score			
	Intercept			
	Patient-level random effect SD			

Table 5. Distribution of other secondary outcomes by trial arm.

		**6 months follow-up**		**12 months follow-up**	
		**Arm A**	**Arm B**	**Arm A**	**Arm B**
Did you practice sun protection last year?	*Responses*<br><br>Rarely or never<br>Sometimes<br>Often<br>always				
How likely are you to practice sun protection in the coming year?	*Responses*<br><br>Not at all likely<br>Unlikely<br>Neither likely nor unlikely<br>Likely<br>Extremely likely				
Ever used sunbeds	*N (proportion)*				
How many times have you been sunburnt in the last year?	*Responses*<br><br>0<br>1-2<br>3-5<br>More than 5				
Compared with other people, do you think your chances of getting melanoma are lower or higher?	*Responses*<br><br>Much lower<br>Lower<br>Neither higher nor lower<br>Higher<br>Much higher				
What do you think your lifetime risk of melanoma is? (%)	*Mean (SD)*				
Have you downloaded any apps for detecting melanoma?	*Responses*<br><br>*Proportion Yes (N)*				
How often have you used this app?	*Responses*<br><br>Never<br>Once every 6 months<br>Once every 3 months<br>Every month				

Table 6. Melanoma incidence in participating practices and in England

	Melanoma diagnoses 2013 to 2015	Population	Crude melanoma incidence (per 100,000 people per year)	(95% CI)
Intervention practices				
England				

Appendix 1. Protocol deviation, use of other SSM apps, analysis of consent bias, analysis of possible differential attrition

Appendix 1 Table 1. Description of protocol deviations

**Description of protocol deviation**	**Possible impact**	**Treatment group**

Appendix 1 Table 2. Use of other skin-self monitoring (SSM) apps

**SSM App**	**Arm A**		**Arm B**	
	**Use once**	**Use repeatedly**	**Use once**	**Use repeatedly**
app name N (%)				
…				
Any app				

Appendix 1 Figure 1. Histogram of use of any other skin-self monitoring apps (A) in trial arm A (B) in trial arm B (C) overall

Three panel histogram.

Appendix 1 Table 3. Data quality checks by reason

**DQ reason**	**DQ outcome**	**Affected (%)**	**Changes to dataset**
Practices with no consultations	Error detected	X practices (%)	X consultations added
Practices with no consultations	Confirmed correct	X practices (%)	No change
Patients with high	Error detected	X patients (%)	X consultations

consultation rates			removed
Patients with high consultation rates	Confirmed correct	X patients (%)	No change

Appendix 1 Table 4. Analysis of possible consent bias. P-values come from two-sample t-tests unless noted otherwise.

		**Declined participation**	**Consented**	**p**
People	*N*			NA
Age	*mean (SD)*			
Female	*proportion*			**

* Chi-squared test

Appendix 1 Table 5. Analysis of non-response. P-values come from two-sample t-tests unless noted otherwise.

**Baseline characteristic**		**Responded to either survey**	**Failed to respond to both surveys**	**P-value**
Pre-trial consultation rate	*mean (SD)*			
Age at randomisation	*mean (SD)*			
Female	*N (proportion)*			*
Social factors inventory 12, Physical component summary	*mean (SD)*			
Social factors inventory 12, Mental component summary	*mean (SD)*			
Sun protection habits score	*mean (SD)*			
Melanoma worry scale	*mean (SD)*			
Self-efficacy for consulting without delay	*mean (SD)*			
HADS-D Depression score	*mean (SD)*			
HADS-A Anxiety score	*mean (SD)*			
Skin self examination benefits score	*mean (SD)*			
Skin self-examination barriers score	*mean (SD)*			
Did you practice sun protection last year?	Rarely or never<br><br>Sometimes<br>Often<br>always			*
How likely are you to practice sun protection in the coming year?	Not at all likely<br><br><br>Unlikely<br>Neither likely nor unlikely			*

**Baseline characteristic**		**Responded to either survey**	**Failed to respond to both surveys**	**P-value**
	Likely<br>Extremely likely			
Ever used sunbeds	*N (proportion)*			
How many times have you been sunburnt in the last year?	0<br><br><br>1-2<br>3-5<br>More than 5			*
Compared with other people, do you think your chances of getting melanoma are lower or higher?	Much lower<br><br><br><br><br>Lower<br>Neither higher nor lower<br>Higher<br>Much higher			*
What do you think your lifetime risk of melanoma is? (%)	*Mean (SD)*			
Have you downloaded any apps for detecting melanoma?	*Proportion Yes (N)*			*
How often have you used this app?	Never<br><br>Once every 6 months<br>Once every 3 months<br>Every month			*

* Chi-squared test

Appendix 2. Additional results relating to outcomes

Appendix 2 Figure 1. Histogram of pre-trial and during-trial consultation rates, overall and by group

Six panel histogram.

Appendix 2 Figure 2. Histogram of patient intervals, overall and by group

Three panel histogram

Appendix 2 Table 1. Descriptive statistics on pre-trial and during-trial consultation rates and during-trial patient intervals, overall and by group

		**Overall**	**Arm A**	**Arm B**
Pre-trial consultation rate	*mean (SD)*			
	median (IQR)			
During-trial consultation rate	*mean (SD)*			
	median (IQR)			
During-trial patient interval	*mean (SD)*			
	median (IQR)			

Appendix 2 Table 2. Results of main and sensitivity analyses of consultation rate during the trial

**Model**	**Factor**	**IRR**	**(95% CI)**	**p-value**
**Main**	*Pearson overdispersion:*			
	Intervention			
	Intercept			
**Sensitivity 1**	*Pearson overdispersion:*			
	Intervention			
	Female gender			
	Age* (years)			
	Intercept			
	GP random effect SD			
**Sensitivity 2**	*Pearson overdispersion:*			
	Intervention			
	Female gender			
	Age* (years, under 65)			
	Age* (years, over 65)			
	Intercept			
	GP random effect SD			

* centered at 65 years

Appendix 2 Table 3. Results of main and sensitivity analyses of patient interval during the trial

Model	Factor	Mean difference	(95% CI)	p-value
**Main**				
	Intervention			
	Intercept			
	Patient-level random effect SD			
**Sensitivity 1**				
	Intervention			
	Female gender			
	Age* (years)			
	Intercept			
	Patient-level random effect SD			
**Sensitivity 2**				
	Intervention			
	Female gender			
	Age* (years, under 65)			
	Age* (years, over 65)			
	Intercept			
	Patient-level random effect SD			

* centered at 65 years

Appendix 2 Table 4. Mean responses on secondary outcome scales overall and by trial arm, from both questionnaires and in each questionnaire individually

Outcome	Follow-up questionnaire	Overall		Arm A		Arm B	
		Mean	(SD)	Mean	(SD)	Mean	(SD)
Social factors inventory 12, Physical component summary	Both						
	6 month						
	12 month						
Social factors inventory 12, Mental component summary	Both						
	6 month						
	12 month						
Sun protection habits score	Both						
	6 month						
	12 month						
Melanoma worry scale	Both						
	6 month						
	12 month						
Self-efficacy for consulting without delay	Both						
	6 month						
	12 month						
HADS-D Depression score	Both						
	6 month						
	12 month						
HADS-A Anxiety score	Both						
	6 month						
	12 month						
Skin self examination benefits score	Both						
	6 month						
	12 month						
Skin self-	Both						

**Outcome**	**Follow-up questionnaire**	**Overall**		**Arm A**		**Arm B**	
		**Mean**	**(SD)**	**Mean**	**(SD)**	**Mean**	**(SD)**
**examination barriers score**							
	**6 month**						
	**12 month**						

Appendix 2 Table 5. Results of sensitivity regression analysis of secondary outcome scales

Outcome	Coefficient	Mean difference	(95% CI)	p-value
**Social factors inventory 12, Physical component summary**				
	Intervention			
	Baseline score			
	Female gender			
	Age* (year)			
	Intercept			
	Patient-level random effect SD			
**Social factors inventory 12, Mental component summary**				
	Intervention			
	Baseline score			
	Female gender			
	Age* (year)			
	Intercept			
	Patient-level random effect SD			
**Sun protection habits score**				
	Intervention			
	Baseline score			
	Female gender			
	Age* (year)			
	Intercept			
	Patient-level random effect SD			
**Melanoma worry scale**				
	Intervention			
	Baseline score			
	Female gender			
	Age* (year)			
	Intercept			
	Patient-level random effect SD			
**Self-efficacy for consulting without delay**				
	Intervention			
	Baseline score			
	Female gender			
	Age* (year)			
	Intercept			
	Patient-level random effect SD			

Outcome	Coefficient	Mean difference	(95% CI)	p-value
**HADS-D Depression score**				
	Intervention			
	Baseline score			
	Female gender			
	Age* (year)			
	Intercept			
	Patient-level random effect SD			
**HADS-A Anxiety score**				
	Intervention			
	Baseline score			
	Female gender			
	Age* (year)			
	Intercept			
	Patient-level random effect SD			
**Skin self examination benefits score**				
	Intervention			
	Baseline score			
	Female gender			
	Age* (year)			
	Intercept			
	Patient-level random effect SD			
**Skin self-examination barriers score**				
	Intervention			
	Baseline score			
	Female gender			
	Age* (year)			
	Intercept			
	Patient-level random effect SD			

***** centered at 65 years

Appendix 3. Additional information on missing data.

Appendix 3 Table 1. Item-level non-response for each secondary outcome, overall and by trial arm

**Outcome scale**	**Question**	**Overall**		**Arm A**		**Arm B**	
		**Possible**	**Missing (proportion)**	**Possible**	**Missing (proportion)**	**Possible**	**Missing (proportion)**
…							
…							

506

507

APPENDIX B. SCORING RULES FOR THE SURVEY INSTRUMENTS.

question	response	Response score (on the Likert scale)	Question weight
Sun protection habits scale (Glanz et al)			
1.1.1. Uses SPF30+ sunscreen	Rarely or never	1	1/4
1.1.1. Uses SPF30+ sunscreen	Sometimes	2	1/4
1.1.1. Uses SPF30+ sunscreen	Often	3	1/4
1.1.1. Uses SPF30+ sunscreen	Always	4	1/4
1.1.2. Wear a hat	Rarely or never	1	1/4
1.1.2. Wear a hat	Sometimes	2	1/4
1.1.2. Wear a hat	Often	3	1/4
1.1.2. Wear a hat	Always	4	1/4
1.1.3 wear a top	Rarely or never	1	1/4
1.1.3 wear a top	Sometimes	2	1/4
1.1.3 wear a top	Often	3	1/4
1.1.3 wear a top	Always	4	1/4
1.1.4 stay in shade	Rarely or never	1	1/4
1.1.4 stay in shade	Sometimes	2	1/4
1.1.4 stay in shade	Often	3	1/4
1.1.4 stay in shade	Always	4	1/4
1.1.5 wear sunglasses	Rarely or never	1	1/4
1.1.5 wear sunglasses	Sometimes	2	1/4
1.1.5 wear sunglasses	Often	3	1/4
1.1.5 wear sunglasses	Always	4	1/4
ANALYSE INDIVIDUALLY			
1.2 Practice sun protection past year	Rarely or never		
1.2 Practice sun protection past year	Sometimes		
1.2 Practice sun protection past year	Often		
1.2 Practice sun protection past year	Always		
1.3 Practice sun protection coming year	Not at all likely		
1.3 Practice sun protection coming year	unlikely		
1.3 Practice sun protection coming year	Neither likely nor unlikely		
1.3 Practice sun protection coming year	likely		
1.3 Practice sun protection coming year	Extremely likely		
1.4 ever used sunbeds	Yes		
1.4 ever used sunbeds	No		
1.5 sunburn in last year	0		
1.5 sunburn in last year	1		
1.5 sunburn in last year	3-5		
1.5 sunburn in last year	More than 5		
Skin Self-examination benefits and barriers scale (Manne & Lessin)			
BENEFITS			
1.6 doing SSE	Strongly disagree	1	1
1.6 doing SSE	Disagree	2	1
1.6 doing SSE	Neither agree nor disagree	3	1
1.6 doing SSE	Agree	4	1
1.6 doing SSE	Strongly agree	5	1
1.7 good health care	Strongly disagree	1	1
1.7 good health care	Disagree	2	1
1.7 good health care	Neither agree nor disagree	3	1
1.7 good health care	Agree	4	1
1.7 good health care	Strongly agree	5	1

1.8 doctor said	Strongly disagree	1	1
1.8 doctor said	Disagree	2	1
1.8 doctor said	Neither agree nor disagree	3	1
1.8 doctor said	Agree	4	1
1.8 doctor said	Strongly agree	5	1
1.9 long life	Strongly disagree	1	1
1.9 long life	Disagree	2	1
1.9 long life	Neither agree nor disagree	3	1
1.9 long life	Agree	4	1
1.9 long life	Strongly agree	5	1
1.10 people close to me	Strongly disagree	1	1
1.10 people close to me	Disagree	2	1
1.10 people close to me	Neither agree nor disagree	3	1
1.10 people close to me	Agree	4	1
1.10 people close to me	Strongly agree	5	1
1.11 regular SSE	Strongly disagree	1	1
1.11 regular SSE	Disagree	2	1
1.11 regular SSE	Neither agree nor disagree	3	1
1.11 regular SSE	Agree	4	1
1.11 regular SSE	Strongly agree	5	1
1.12 piece of mind	Strongly disagree	1	1
1.12 piece of mind	Disagree	2	1
1.12 piece of mind	Neither agree nor disagree	3	1
1.12 piece of mind	Agree	4	1
1.12 piece of mind	Strongly agree	5	1
BARRIERS			
1.13 not confident	Strongly disagree	1	1
1.13 not confident	Disagree	2	1
1.13 not confident	Neither agree nor disagree	3	1
1.13 not confident	Agree	4	1
1.13 not confident	Strongly agree	5	1
1.14 too many moles	Strongly disagree	1	1
1.14 too many moles	Disagree	2	1
1.14 too many moles	Neither agree nor disagree	3	1
1.14 too many moles	Agree	4	1
1.14 too many moles	Strongly agree	5	1
1.15 nervous	Strongly disagree	1	1
1.15 nervous	Disagree	2	1
1.15 nervous	Neither agree nor disagree	3	1
1.15 nervous	Agree	4	1
1.15 nervous	Strongly agree	5	1
1.16 anxious	Strongly disagree	1	1
1.16 anxious	Disagree	2	1
1.16 anxious	Neither agree nor disagree	3	1
1.16 anxious	Agree	4	1
1.16 anxious	Strongly agree	5	1
1.17 embarrassing	Strongly disagree	1	1
1.17 embarrassing	Disagree	2	1
1.17 embarrassing	Neither agree nor disagree	3	1
1.17 embarrassing	Agree	4	1
1.17 embarrassing	Strongly agree	5	1
1.18 staying out of the sun	Strongly disagree	1	1
1.18 staying out of the sun	Disagree	2	1
1.18 staying out of the sun	Neither agree nor disagree	3	1
1.18 staying out of the sun	Agree	4	1

1.18 staying out of the sun	Strongly agree	5	1
1.19 gets in the way	Strongly disagree	1	1
1.19 gets in the way	Disagree	2	1
1.19 gets in the way	Neither agree nor disagree	3	1
1.19 gets in the way	Agree	4	1
1.19 gets in the way	Strongly agree	5	1
1.20 too much time	Strongly disagree	1	1
1.20 too much time	Disagree	2	1
1.20 too much time	Neither agree nor disagree	3	1
1.20 too much time	Agree	4	1
1.20 too much time	Strongly agree	5	1
1.21 prefer doctor	Strongly disagree	1	1
1.21 prefer doctor	Disagree	2	1
1.21 prefer doctor	Neither agree nor disagree	3	1
1.21 prefer doctor	Agree	4	1
1.21 prefer doctor	Strongly agree	5	1
1.22 difficult	Strongly disagree	1	1
1.22 difficult	Disagree	2	1
1.22 difficult	Neither agree nor disagree	3	1
1.22 difficult	Agree	4	1
1.22 difficult	Strongly agree	5	1
Melanoma worry scale (Moye et al)			
2.1 worry about melanoma	Not at all	1	1
2.1 worry about melanoma	Rarely	2	1
2.1 worry about melanoma	Sometimes	3	1
2.1 worry about melanoma	Often	4	1
2.1 worry about melanoma	Almost all the time	5	1
2.2 worries affecting your mood	Not at all	1	1
2.2 worries affecting your mood	A little	2	1
2.2 worries affecting your mood	Somewhat	3	1
2.2 worries affecting your mood	A lot	4	1
2.3 worries affecting daily activities	Not at all	1	1
2.3 worries affecting daily activities	A little	2	1
2.3 worries affecting daily activities	Somewhat	3	1
2.3 worries affecting daily activities	A lot	4	1
2.4 anxiety about results	Not at all	1	1
2.4 anxiety about results	A little	2	1
2.4 anxiety about results	Somewhat	3	1
2.4 anxiety about results	A lot	4	1
Perceived melanoma risk (Manne & Lessin) ANALYSE INDIVIDUALLY			
2.5 changes for melanoma	Much lower	1	
2.5 changes for melanoma	Lower	2	
2.5 changes for melanoma	Neither higher or nor lower	3	
2.5 changes for melanoma	Higher	4	
2.5 changes for melanoma	Much higher	5	
2.6 lifetime risk	0-100% range	0-100% range	
Self-efficacy for consulting without delay ANALYSE INDIVIDUALLY			
2.7 usual doctor	1-10 scale	1-10	1
2.8 phone number Is busy	1-10 scale	1-10	1
2.9 time away	1-10 scale	1-10	1
2.10 not serious	1-10 scale	1-10	1
2.11 haven't seen before	1-10 scale	1-10	1
2.12 still worried	1-10 scale	1-10	1
2.13 unusual colour	1-10 scale	1-10	1
2.14 not offered within 3 days	1-10 scale	1-10	1

Apps (I don't think this is a validated questionnaire as such) ANALYSE INDIVIDUALLY			
3.1a downloaded any apps?	No		
	Yes		
3.1b if yes, name	Freetext		
3.1c how often used?	Never		
3.1c how often used?	Once		
3.1c how often used?	One every 6 months		
3.1c how often used?	Once every 3 months		
3.1c how often used?	Every month		
Hospital anxiety and depression scale (Zigmund & Snaith)			
4.1 wound up	Most of the time	3 A (anxiety)	1
	A lot of the time	2 A (anxiety)	1
	Time to time, occasionally	1 A (anxiety)	1
	Not at all	0 A (anxiety)	1
4.2 slowed down	Nearly all of the time	3 A (anxiety)	1
	Very often	2 A (anxiety)	1
	Sometimes	1 A (anxiety)	1
	Not at all	0 A (anxiety)	1
4.3 enjoy things	Definitely as much	0 D (depression)	1
	Not quite so much	1 D (depression)	1
	Only a little	2 D (depression)	1
	Not at all	3 D (depression)	1
4.4 butterflies	Not at all	0 D (depression)	1
	Occasionally	1 D (depression)	1
	Quite often	2 D (depression)	1
	Very often	3 D (depression)	1
4.5 awful	Very definitely and quite badly	3 A (anxiety)	1
	Yes, but not too badly	2 A (anxiety)	1
	A little, but it doesn't bother me	1 A (anxiety)	1
	Not at all	0 A (anxiety)	1
4.6 appearance	Definitely	3 A (anxiety)	1
	I don't take as much care as I should	2 A (anxiety)	1
	I may not quite take as much care	1 A (anxiety)	1
	I take just as much care as ever	0 A (anxiety)	1
4.7 laugh	As much as I always could	0 D (depression)	1
	Not quite so much now	1 D (depression)	1
	Definitely not so much now	2 D (depression)	1
	Not at all	3 D (depression)	1
4.8 restless	Very much indeed	3 A (anxiety)	1
	Quite a lot	2 A (anxiety)	1
	Not very much	1 A (anxiety)	1
	Not at all	0 A (anxiety)	1
4.9 worrying	A great deal of the time	3 A (anxiety)	1
	A lot of the time	2 A (anxiety)	1
	From time to time but not too often	1 A (anxiety)	1
	Only occasionally	0 A (anxiety)	1
4.10 enjoyment	As much as I ever did	0 D (depression)	1
	Rather less than I used to	1 D (depression)	1
	Definitely less than I used to	2 D (depression)	1
	Hardly at all	3 D (depression)	1
4.11 cheerful	Not at all	3 A (anxiety)	1
	Not often	2 A (anxiety)	1
	Sometimes	1 A (anxiety)	1
	Most of the time	0 A (anxiety)	1
4.12 panic	Very often indeed	3 A (anxiety)	1

	Quite often	2 A (anxiety)	1
	Not very often	1 A (anxiety)	1
	Not at all	0 A (anxiety)	1
4.13 relaxed	Definitely	0 D (depression)	1
	Usually	1 D (depression)	1
	Not often	2 D (depression)	1
	Not at all	3 D (depression)	1
4.14 Enjoy book	Often	0 D (depression)	1
	Sometimes	1 D (depression)	1
	Not often	2 D (depression)	1
	Very seldom	3 D (depression)	1
SF-12 quality of life scale – REFER TO REFERENCES FOR SCORING RULESA			
4.15 general health	Excellent		
	Very good		
	Good		
	Fair		
	poor		
4.16 health limit	Yes, limited a lot		
	Yes, limited a little		
	No, not limited at all		
4.17 climbing stairs	Yes, limited a lot		
	Yes, limited a little		
	No, not limited at all		
4.18 accomplished	Yes		
	No		
4.19 kind of work	Yes		
	No		
4.20 emotional accomplished	Yes		
	No		
4.21 careful	Yes		
	No		
4.22 pain	Not at all		
	A little bit		
	Moderately		
	Quite a bit		
	extremely		
4.23 calm and peaceful	All of the time		
	Most of the time		
	A good bit of the time		
	Some of the time		
	A little of the time		
	None of the time		
4.24 lots of energy	All of the time		
	Most of the time		
	A good bit of the time		
	Some of the time		
	A little of the time		
	None of the time		
4.25 downhearted and blue	All of the time		
	Most of the time		
	A good bit of the time		
	Some of the time		
	A little of the time		
	None of the time		
4.26 social activities	All of the time		

	Most of the time		
	A good bit of the time		
	Some of the time		
	A little of the time		
	None of the time		
