# Supplementary material for: Effect of a Skin Self-monitoring Smartphone Application on Time to Physician Consultation Among Patients With Possible Melanoma: A Phase 2 Randomized Clinical Trial
Source: JAMA Netw Open. 2020 Feb 26;3(2):e200001. doi: 10.1001/jamanetworkopen.2020.0001 (PMC7137684; doi:10.1001/jamanetworkopen.2020.0001)
Supplement: Supplement 3. — Data Sharing Statement [file jamanetwopen-3-e200001-s003.pdf]

Data Sharing Statement

Walter. Effect of a Skin Self-monitoring Smartphone Application on Time to Physician Consultation Among Patients With Possible Melanoma. *JAMA Netw Open*. Published February 26, 2020. 10.1001/jamanetworkopen.2020.0001

Data
Data available: Yes
Data types: Deidentified participant data
How to access data: Upon request to authors, via fmw22@medschl.cam.ac.uk
When available: With publication

Supporting Documents
Document types: None

Additional Information
Who can access the data: Anyone requesting data
Types of analyses: for a specified purpose
Mechanisms of data availability: with a signed data access agreement
Any additional restrictions: none
